# Exploring Diverse-Ring Analogues on Combretastatin A4 (CA-4) Olefin as Microtubule-Targeting Agents

**DOI:** 10.3390/ijms21051817

**Published:** 2020-03-06

**Authors:** Ming-Yu Song, Qiu-Rui He, Yi-Lin Wang, Hao-Ran Wang, Tian-Cheng Jiang, Jiang-Jiang Tang, Jin-Ming Gao

**Affiliations:** 1 Shaanxi Key Laboratory of Natural Products & Chemical Biology, College of Chemistry & Pharmacy, Northwest A&F University, Yangling 712100, China; mingyusong@nwsuaf.edu.cn (M.-Y.S.); heqiurui@nwsuaf.edu.cn (Q.-R.H.); jinminggao@nwsuaf.edu.cn (J.-M.G.); 2 College of Innovation and Experiment, Northwest A&F University, Yangling 712100, China; 3 College of Food Science and Engeering, Northwest A&F University, Yangling 712100, China

**Keywords:** combretastatin-4, tubulin polymerization, diphenylethanone, *cis*-locked dihydrofuran, α-substituted diphenylethanone, cyclobutane, cyclohexane

## Abstract

Combretastatin-4 (CA-4) as a tubulin polymerization inhibitor draws extensive attentions. However, due to its weak stability of *cis*-olefin and poor metabolic stability, structure modifications on *cis*-configuration are being performed. In this work, we constructed a series of novel CA-4 analogues with linkers on olefin containing diphenylethanone, *cis*-locked dihydrofuran, α-substituted diphenylethanone, cyclobutane and cyclohexane on its *cis*-olefin. Cytotoxic activity of all analogues was measured by an SRB assay. Among them, compound **6b**, a by-product in the preparation of diphenylethanone analogues, was found to be the most potent cytotoxic agents against HepG2 cells with IC_50_ values of less than 0.5 μM. The two isomers of **6b** induced cellular apoptosis tested by Annexin V-FITC and propidium iodide (PI) double staining, arrested cells in the G2/M phase by PI staining analysis, and disrupted microtubule network by immunohistochemistry study in HepG2 cells. Moreover, **6b-(*E*)** displayed a dose-dependent inhibition effect for tubulin assembly in in vitro tubulin polymerization assay. In addition, molecular docking studies showed that two isomers of **6b** could bind efficiently at colchicine binding site of tubulin similar to CA-4.

## 1. Introduction

**Figure 1:**
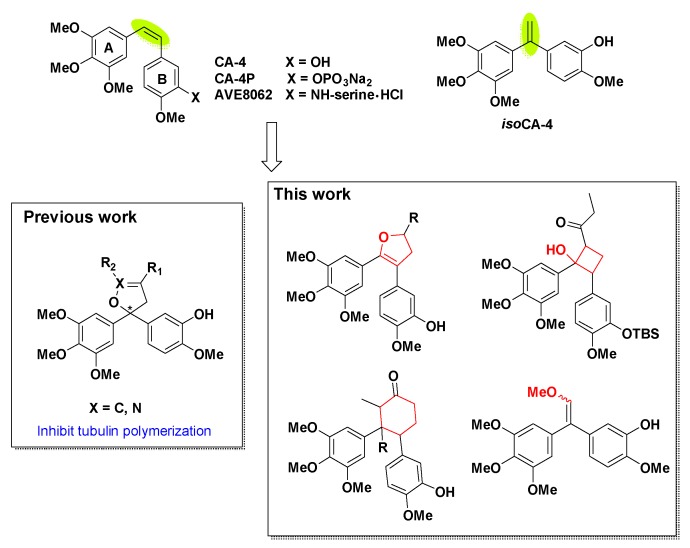
The structures of CA-4 and synthetic prodrugs, *iso*CA-4 and designed analogues.

The natural product combretastatin A-4 (CA-4, Figure 1) was first isolated from the South African tree *Combretum caffrum* in 1989 [1,2] and was serendipitously discovered in 1998 by strongly blocking the polymerization of tubulin with the colchicine binding site [3]. CA-4 and its water-soluble prodrugs such as CA-4P (fosbretabulin and zybrestat) [4] and AVE8062 (ombrabulin) [5] have entered clinical trials for the treatment of anaplastic thyroid cancer and other solid tumors in both USA and Europe [6]. Due to its small molecular weight and simple structure, an impressive number of synthetic research was carried out over the past decades [7,8,9,10].

The structure–activity relationships (SARs) have made clearly important features of maintaining the tubulin interaction for CA-4. The presence of 3,4,5-trimethoxy groups at ring-A, 4‘-methoxy group at ring-B and *cis*-configuration of olefin are the indispensability for inhibiting tubulin assembly [11]. Nevertheless, the *Z*-restricted configuration can rapidly convert into 100-fold less active *trans*-isomerization under the conditions of suffering from heat, light and protic media [12]. Meanwhile, the poor metabolic stability caused by the olefinic bridge and the weak water solubility of CA-4 resulted in restrictions for its exploitation [13]. Thus, an extensive focus of research was directed towards the rational modifications of the unstable olefinic bond as the main pursued strategy. Various olefinic linkers including silicon [14], butadiene [15], cyclopropane [16], cyclopropyl [17], chiral-lactam [18], furan [19], furazan [20], imidazole [21], 2,3-diarylthiophene [22], *iso*xazole and terphenyl [23], triazoles [24,25], thiazole [26,27] and 1,2,4-triazolo-4,3-*b*-pyridazines [28] has unveiled novel agents endowed with cytotoxic activity.

*Iso*combretastatin A-4 (*iso*CA-4, Figure 1), an unexpectedly synthesized isomer of CA-4 found by Alami [29], exhibited a very strong inhibition on cancer cell growth similar to that of CA-4. The synthetic method is simple without the control of *cis*-configuration [30,31]. The Alami group have completed plentiful studies on the modification of 1,1-diaryl double bond scaffold using naphthenic [32], naphthalene [33], arylchromenes [34], azaisoerianin [35], arylbenzoxepins [36], etc. In our previous works [37], dihydrofuran and dihydroisoxazole analogues of *iso*CA-4 were prepared through [3+2] reactions and could inhibit tubulin polymerization. 

In our endeavor aiming at finding novel potential rigid analogues of CA-4 and *iso*CA-4, we hypothesized that the replacement of the unstable olefinic bond of CA-4 with *cis*-locked dihydrofuran, α-substituted, cyclobutane and cyclohexane nucleuses (Figure 1) could be a good strategy to unravel a novel class of antitubulin agents. Indeed, different number rings are widely used as a molecular scaffold in natural products and medicinal chemistry because of their favorable pharmacokinetic features and their ability to act as a privileged structure when properly decorated. In this work, we present the synthesis through the modification of olefin scaffold with diphenylethanone, *cis*-locked dihydrofuran, α-substituted diphenylethanone, cyclobutane and cyclohexane. Subsequently, their cytotoxic activity was evaluated against HepG2 cells, and the most potent cytotoxic compound was selected to carry out mechanism studies including apoptosis, cell cycle analysis, immunohistochemistry, tubulin polymerization assay and molecular docking.

## 2. Results and Discussion

### 2.1. Chemistry

**Scheme 1:**
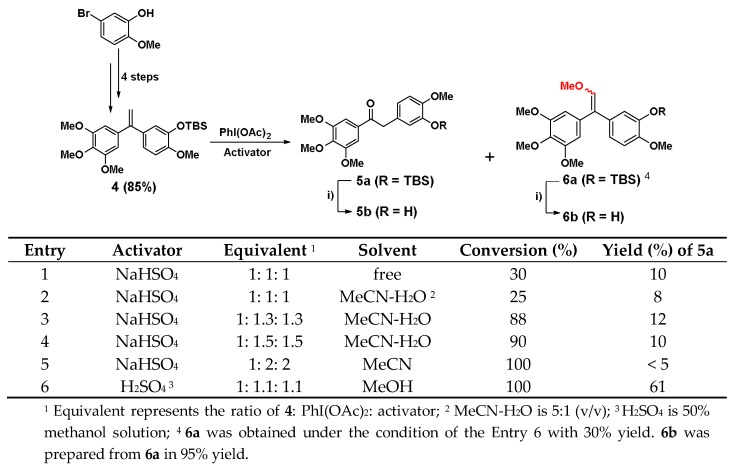
Synthesis of compounds **5a**, **5b** and **6a**, **6b**. Synthetic conditions optimization of **5a**. i) TBAF, THF, 30 min.

**Figure 2:**
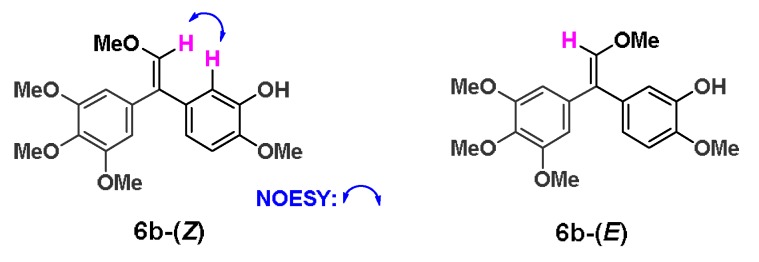
**6b-(*Z*)** and **6b-(*E*)** after HPLC preparation. Selected NOE correlations of **6b-(*Z*)**.

5-bromo-2-methoxyphenol was firstly converted into the key intermediate **4** by four steps in total 65% yield referred to our previous report [37]. Subsequently, **4** was oxidized with rearrangement of olefinic bond through iodobenzene acetate (PhI(OAc)_2_) to afford the key compound **5a**. The synthetic conditions were optimized in Scheme 1. In the synthesis of **5a**, NaHSO_4_ was used as the initial activator of PhI(OAc)_2_. However, the conversion of **4** was always very low in the presence or absence of solvent as shown in entry 1 and 2, and the yield of **5a** was only about 10% when the ratio of **4**, PhI(OAc)_2_ and NaHSO_4_ was 1:1:1. As the ratio of PhI(OAc)_2_ and NaHSO_4_ continued to increase, the conversion rate of **4** was indeed greatly improved when the reaction adopted MeCN-H_2_O (5:1) as the solvent in entry 3 and 4. When both of PhI(OAc)_2_ and NaHSO_4_ reached 2 equivalent (entry 5), **4** could react completely, but the yield of **5a** was still only about 10% or even lower. After the NaHSO_4_ was replaced with H_2_SO_4_ (50% methanol solution), the reaction was completed with a 61% yield at -20 °C, while the ratio of **4**, PhI(OAc)_2_, and H_2_SO_4_ was 1:1.1:1.1 in entry 6. Accidentally, a by-product **6a** was given under the condition of entry 6 with 30% yield, and **6b** was prepared from **6a** using tetrabutyl ammonium fluoride (TBAF) to remove the protection group in 95% yield. To the formation of the by-product **6a**, MeOH may attack the intermediate of PhI(OAc)_2_ and H_2_SO_4_ in reaction kinetics and through following elimination reaction to trigger the formation of **6a**. The possible reaction mechanism was shown as Scheme S1 in the Supplementary Material. Furthermore, the HPLC analysis and ^13^C NMR spectrum of **6b** revealed that the ratio of (*Z*)*/*(*E*) isomers was about 1:1. To explore biological activities of different isomers, **6b-(*Z*)** and **6b-(*E*)** were separated by preparative HPLC. The assignment of the olefin geometry of **6b-(*Z*)** was determined using NOESY NMR experiments. Specifically, NOESY signals of **6b-(*Z*)** were readily apparent between the proton of phenyl ring and the proton attached to the olefin in Figure 2 (see Supplementary data).

**Scheme 2:**
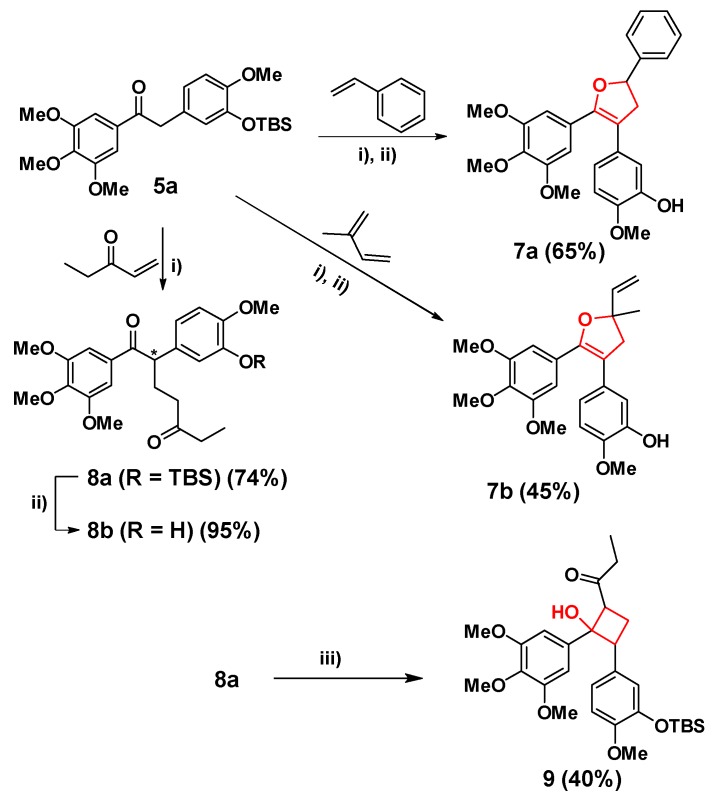
Synthesis of **7a, 7b, 8a, 8b** and **9**. Condition: i) Cu(OAc)_2_, DCE, 110 °C, 4 h; ii) TBAF, THF, 0.5 h; iii) LiHMDS, TsCl, −78 °C.

With **5a** in hands, dihydrofuran analogues were prepared by [3 + 2] cycloaddition in Scheme 2. A free-radical reaction between **5a** and conjugated diolefinic compounds was catalyzed by a single-electron oxidant Cu(OAc)_2_, and following by TBAF to remove the TBS group of the phenolic hydroxyl to provide the dihydrofuran **7a** and **7b**. In this free-radical reaction, Mn(OAc)_3_ as the initial oxidant gave extremely low yield, perhaps the single-electron oxidation energy of Mn (III) (1.5 V) could still be too high for this reaction. Thus, Cu (II) with one-electron oxidation energy of only 0.16 V was employed for this reaction and the conditions were optimized by using Cu(OAc)_2_ as the catalyst and the acetophenone as the conjugated olefin. It was found that when the ratio of **5a**, Cu(OAc)_2_ and acetophenone was 1:3:3, **5a** could react completely in 12 h. However, a large amount of by-products would appear after 4 h. Therefore, the optimal reaction time should be 4 h, while the conversion rate of **5a** was about 70%, and the yield of **7a** was 65%. Using this condition, dihydrofuran compound **7b** was also synthesized using isoprene, and the two steps yield was 45%. 

However, under the above condition, an addition reaction between **5a** and pentenone occurred to give compound **8a** with a conversion yield of 74% (Scheme 2). The reason that **8a** did not cyclize into a dihydrofuran ring might be the electron-withdrawing effect of the carbonyl group. **8b** was afforded by deprotection of the TBS group similar to the above method. Next, the structure of 1,5-dione of **8a** gave us a new idea to synthesize cyclobutane and cyclohexane analogues of CA-4 through Aldol condensation. For example, cyclobutane analogue **9** was prepared under the conditions of LiHMDS and TsCl at −78 °C in a 40% yield (Scheme 2).

For synthesis of cyclohexane analogues, the Aldol condensation of **8a** was not smooth due to the not very stable TBS protecting group under a strong base condition. On the one hand, strong alkalinity can promote the reaction to proceed forward. However, on the other hand, it would cause the removal of TBS under the action of too strong bases, then reduce the number of substrate and push the reverse reaction. Therefore, it is very important to choose a base with moderate alkalinity. 

**Scheme 3:**
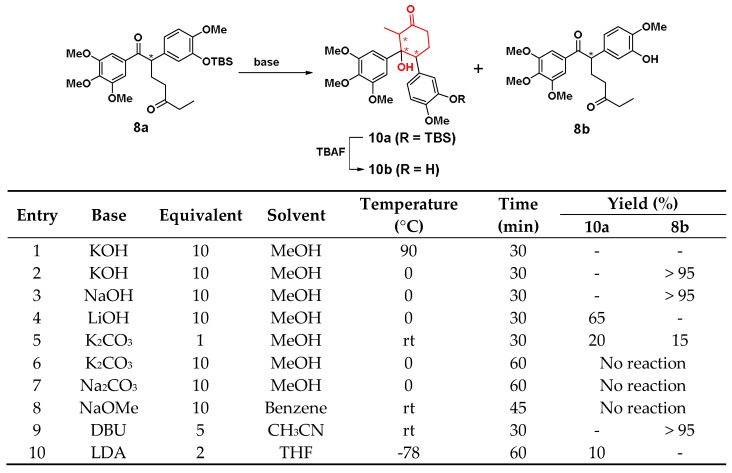
Synthetic conditions optimization of compound **10a**.

The condition optimization of Aldol condensation is shown in Scheme 3. At first, the reaction was performed with KOH in methanol under reflux, **8a** was completely converted quickly, but there was no expected product **10a** or **8b** in entry 1. Then, three inorganic bases: KOH, NaOH and LiOH (entry 2-4) were selected and methanol was employed as the solvent at 0 °C. After the three bases were added, the reaction occurred immediately, and **10a** was obtained in the beginning period. However, 10 min later, the reactions of KOH and NaOH began to generate **8b**. After another 20 min, all **10a** and **8a** were turned into **8b**. However, the reaction of LiOH (entry 4) started to generate **8b** after 30 min, while the conversion yield of **10a** is 65%. Under the action of K_2_CO_3_ (1 eq, entry 5), **8a** could rapidly generate a mixture of **10a**, **8b** and a little deprotected product of **10a** at room temperature. Yet, when the temperature fell to 0 °C, **8a** was completely unreactive even increasing the equivalent of K_2_CO_3_ to 10 eq (entry 6). NaOMe also could not make **8a** react in solvent of benzene in entry 8. In organic bases, 1,8-diazabicyclo[5.4.0]undec-7-ene (DBU) could make **8a** to turn into **8b** within 30 min at room temperature, and lithium diisopropylamide (LDA) could give a 10% yield of **10a** at −78 °C, in entry 9 and 10, respectively. To sum up, LiOH, 0 °C, and 30 min were the optimized conditions with a conversion yield of 65% for **10a**. After **10a** was deprotected by TBAF, a cyclohexane-based CA-4 analogue **10b** was obtained (Scheme 3).

**Scheme 4:**
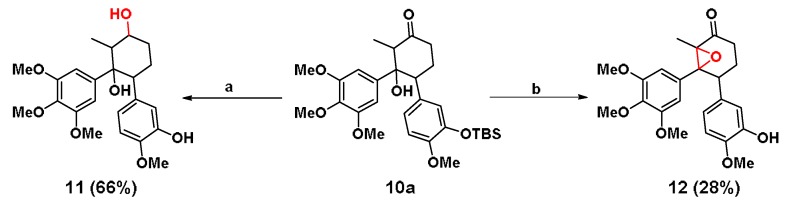
Synthesis of **11** and **12**. Condition: (**a**) i) NaBH_4_, DCE, 0 °C, 1 h; ii) TBAF, THF, 30 min and (**b**) i) LiHMDS, TsCl, -78 °C; ii) Et_3_N, DMAP, DMF, 80 °C and iii) TBAF, THF, 30 min.

In order to enhance the polarity and water solubility of **10b**, carbonyl group of **10a** was reduced by sodium borohydride (NaBH_4_) to a HO group (Scheme 4). After deprotection of the TBS group by TBAF, **11** was obtained in two steps with a 66% yield. Moreover, LiHMDS was employed to remove the hydroxyl hydrogen of **10a**, and then *p*-toluenesulfonyl chloride (TsCl) and the DMAP-Et_3_N system were used to eliminate the sulfonyl group. Finally, the TBS group was deprotected by TBAF to give another cyclohexane analogue **12** (Scheme 4). The total yield of the three steps was 28%. It was noted that **12** was unable to react under the action of NaBH_4_ to afford **11**.

### 2.2. Biological Activity

New synthesized CA-4 analogues were in vitro screened for anticancer activity against HepG-2 (human liver cancer cell line) with CA-4 and *iso*CA-4 as reference drugs using the sulforhodamine B (SRB) assay [38]. The IC_50_ values (the concentration to cause 50% inhibition of cell viability) were summarized in Table 1. It could be seen that these CA-4 analogues diphenylethanone **5b,** *cis*-locked dihydrofuran analogues **7a 7b**, α-substituted diphenylethanones **8a**, **8b,** cyclobutane analogue **9**, cyclohexane analogues **10a**, **10b** and **11** showed weak cytotoxic activity with IC_50_ values over 10 µM. Another cyclohexane analogue **12** exhibited a good cytotoxic activity with IC_50_ of 3.67 µM, but with far less than IC_50_ of CA-4 and *iso*CA-4 (0.013 µM and 0.017 µM, respectively). These results suggested that introductions of ethanone, four or five or six-membered rings on the *cis*-double bond of CA-4 were not a good synthetic strategy for optimizing anticancer activity. However, it was worth to note that *iso*CA-4 analogue **6b** displayed strongest activity with IC_50_ of < 0.5 µM among all compounds. Notably, the IC_50_ results of **6b-(*Z*)** with IC_50_ of 0.30 µM and **6b-(*E*)** with IC_50_ of 0.23 µM on HepG2 cells indicated no obvious gap in cytotoxic activity between *cis–trans* isomers.

**Figure 3:**
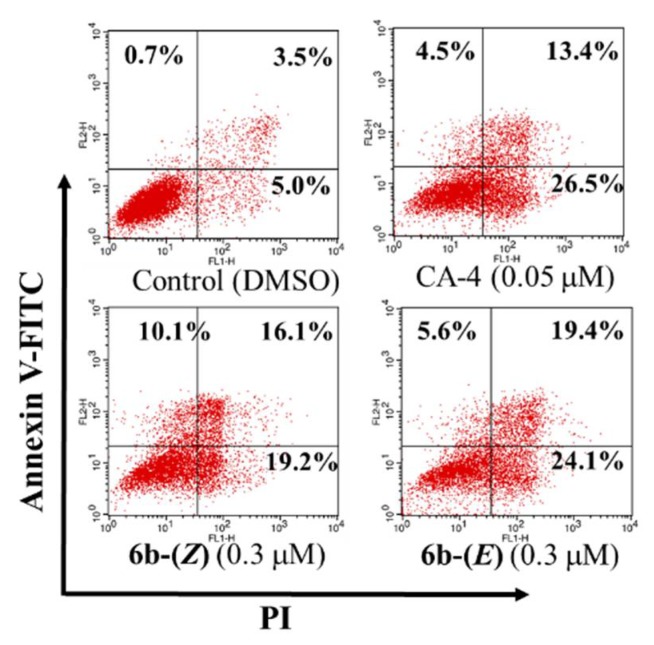
Induction of apoptosis by **6b-(*Z*)**, **6b-(*E*)** and CA-4 at the indicated concentrations on HepG-2 cells. After treatment for 48 h, the cells were stained with PI and Annexin V-FITC and viable, apoptotic and necrotic cells were analyzed by flow cytometry. Cells in lower left quadrants are alive, in the lower right quadrants are in early apoptosis, in the upper right quadrants are in late apoptosis and in the upper left quadrants are necrotic. Percentage of the total signal within the quadrant is indicated. Each experiment was performed in triplicate.

Apoptosis induction is characteristic of many known anticancer agents. To determine whether the inhibition of **6b-(*Z*)** and **6b-(*E*)** on cell growth was related to cell apoptosis, a flow cytometric analysis by staining cells with Annexin V-FITC and propidium iodide (PI) was performed on HepG-2 cells. The result showed that treatment with 0.05 µM CA-4 for 48 h induced 26.5% and 13.4% early and late apoptotic cells, respectively (Figure 3). Similar apoptosis induced by CA-4 has been reported previously [3]. After treatment with compounds **6b-(*Z*)** and **6b-(*E*)** at 0.3 µM, the early and late apoptosis rates were significantly increased from 5.0% and 3.5% (for control group) to 19.2% and 16.1% (for **6b-(*Z*)** group), and 24.1% and 19.4% (for **6b-(*E*)** group), respectively. The results indicated that both analogues could cause cellular apoptosis.

**Figure 4:**
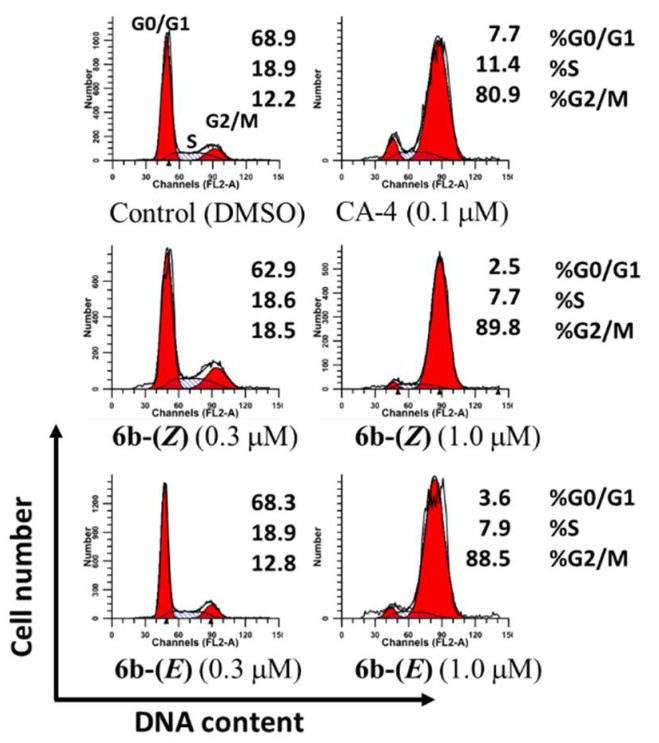
Analysis of cell cycle distribution in HepG-2 cells by flow cytometry. The cultured cells were treated with **6b-(*Z*)**, **6b-(*E*)** and CA-4 at the indicated concentrations for 24 h, then harvested, and analyzed using a FACS Calibur (BD Biosciences).

A striking character of anticancer activity of CA-4 and *iso*CA-4 is to induce cell cycle arrest at the G2/M phase [3,39]. To confirm whether the biological mechanism of these two isomers was caused by cell cycle accumulation at a G2/M phase, the cell cycle distribution of HepG2 cells was evaluated with PI staining by flow cytometry [40]. As shown in Figure 4, following treatment with **6b-(*Z*)** and **6b-(*E*)** for 24 h at concentrations of 0.3 µM and 1.0 µM, the percentages of cells in the G2/M phase at different concentrations were 18.5% and 89.8% for **6b-(*Z*)** and 12.8% and 88.5% for **6b-(*E*)**, respectively, as compared to 12.2% in control cells, suggesting that these two isomers caused an obvious G2/M arrest with a similar mechanism to that of CA-4.

**Figure 5:**
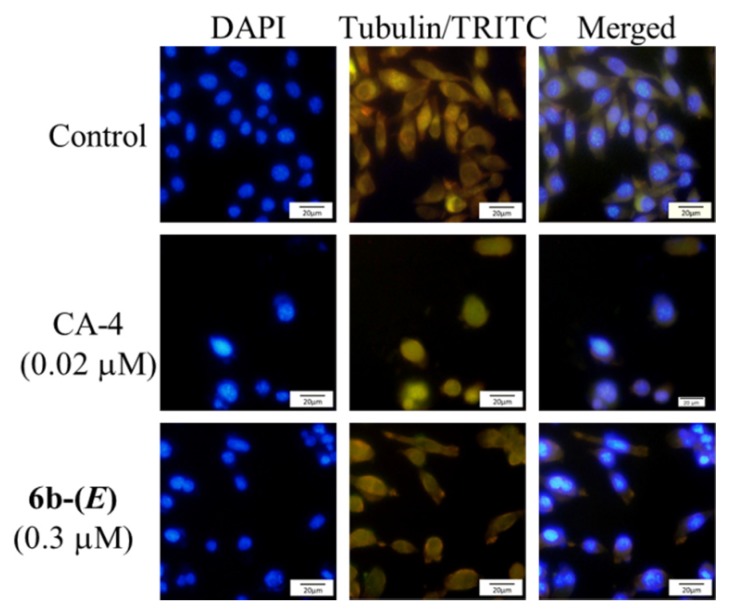
Immunofluorescence effects of **6b-(*E*)** (0.3 μM) and CA-4 (0.02 μM) on inhibition of tubulin polymerization in HepG-2 cells. After treatment for 48 h, the cells were stained with DAPI (blue, left panel) for nucleus and an anti-α-tubulin antibody (TRITC, green, middle panel) for microtubules. The right panel represents a merge of the corresponding left and middle panels. Images were acquired with an Olympus fluorescence microscope using a 20× objective.

To visualize the tubulin polymerization inhibition of these compounds in cells, immunofluorescence analysis was investigated on microtubule network in HepG2 cells. As shown in Figure 5, control cells displayed a normal distribution of microtubular network with a slim and fibrous shape. However, cells treated with CA-4 and **6b-(*E*)** showed a disrupted microtubule network (green) becoming short and wrapped around cell nucleus, demonstrating that **6b-(*E*)** could inhibit the cellular tubulin polymerization.

**Figure 6:**
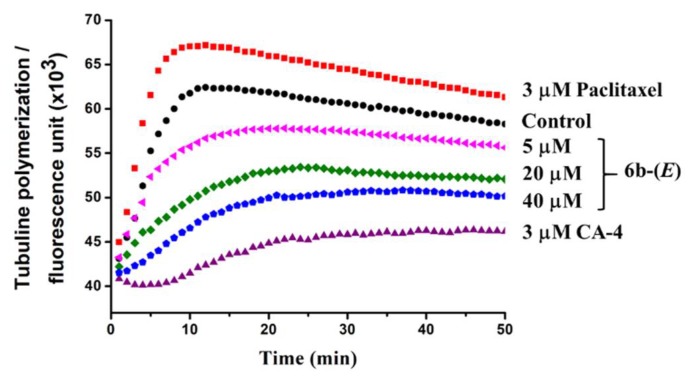
Effects of **6b-(E)** on tubulin polymerization in vitro. Tubulin (10 mg/mL) was incubated with **6g-(E)** (5, 20 and 40 µM) for 50 min at 37 °C. Paclitaxel (3 µM) and CA-4 (3 µM) were used as positive controls. The fluorescence intensity was monitored every minute for characterization of tubulin polymerization at a 360 nm excitation wavelength and 460 nm emission wavelength.

Furthermore, to investigate whether there is such an interaction of the active analogues with tubulin responsible for the activity, tubulin polymerization in vitro was measured for 50 min at 37 °C with paclitaxel as a promoting-assembly control and CA-4 as a depolymerization control. As shown in Figure 6, paclitaxel promoted the assembly of tubulin, while CA-4 inhibited it well. Compound **6b-(*E*)** displayed an inhibition effect for tubulin polymerization in a dose-dependent manner. From these results, it could be concluded that **6b-(*E*)** could inhibit the assembly of tubulin, and the cellular activities of **6b-(*E*)** might be generated from this interaction with tubulin.

### 2.3. Molecular Docking

**Figure 7:**
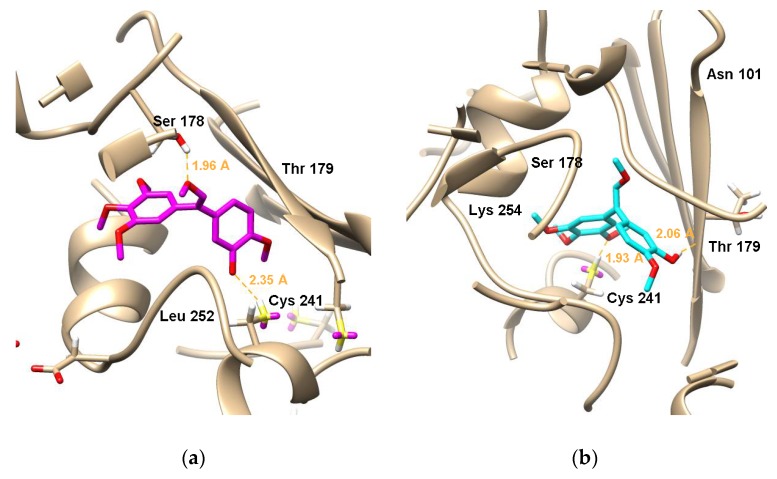
Molecular docking simulations obtained at lowest energy conformation, highlighting potential hydrogen contacts of (**a**) **6b-(*Z*)** and (b) **6b-(*E*)** with the colchicine’s binding site of tubulin (PDB code: 1SA0). Surrounding key amino acid residues are labeled, and hydrogen bonds are shown by orange dashed lines.

To elucidate the binding model of the analogues **6b-(*Z*)** and **6b-(*E*)** with the tubulin colchicine binding site, the X-ray crystal structure (PDB:1SA0) was employed for performing the docking studies using the Surflex-Dock program in the Sybyl-X 2.0 software. Figure 7a,b exhibits the docking studies of **6b-(*Z*)** and **6b-(*E*)** with the tubulin colchicine binding site of tubulin, respectively. Figure 7a shows that the *meta*-methoxy groups on alkene of **6b-(*E*)** formed a hydrogen bond with the key amino acid Ser178 about 1.96 Å bond distance, and the oxygen atoms of the hydroxyl groups on ring-B of **6b-(*Z*)** formed a hydrogen bond with the key amino acid Cys241 about 2.35 Å. Figure 7b exhibits that the *meta*-methoxy groups on ring-A of **6b-(*E*)** formed a hydrogen bond with the key amino acid Cys241 about 1.93 Å, while the oxygen atoms of the hydroxyl groups on ring-B formed a hydrogen bond with the key amino acid Thr193 about 2.06 Å. From the simulated bond distance results, it can be concluded that **6b-(*E*)** should have a better biological activity than **6b-(*Z*)**, consisting with cytotoxicity test results. These docking studies suggested that the binding site of **6b-(*Z*)** and **6b-(*E*)** might be the tubulin colchicine binding site.

## 3. Materials and Methods 

### 3.1. Chemistry

#### 3.1.1. General

All the compounds were recorded by a 500 MHz Bruker NMR spectrometer (Bruker Daltonics Inc., Bremen, Germany) with ^1^H, ^13^C and DEPT135 NMR in CDCl_3_ solution with TMS as internal standard for protons. The 2D NOSEY experiment was carried out in the 500 MHz Bruker NMR spectrometer with 32 of the scans number. Chemical shift values are mentioned in *δ* (ppm) and coupling constants (*J*) in Hz. MS (ESI) was measured on an ESI-Thermo Fisher LTQ Fleet instrument spectrometer (Thermo Fisher Scientific Inc., Waltham, MA)The reaction progress was supervised by thin-layer chromatography (TLC), spots were observed under UV light (254 nm and 365 nm) and colorized with 5% phosphomolybdic acid. Silica gels (300-400 mesh, Qingdao Marine Chemical Ltd., Qingdao, China) were used for column chromatography. Waters 1525 series equipped with a PDA detector (Waters Corporation, Milford, MA) was used for analytical HPLC, and YMS-pack ODS-A column (250 mm × 10 mm, 5 µm) was used for semipreparation. All reagents were purchased from commercial sources, and purifications and desiccations of available solvents were accomplished by standard techniques prior to use. Positive control drug CA-4 was purchased from Sigma, and *iso*CA-4 was obtained refer to the previous report [37]. The purity of all compounds was confirmed to be greater than 95% through analytical HPLC.

#### 3.1.2. Synthesis of Diphenylethanone Analogue **5a**, **5b** and by-Products **6a**, **6b**

Preparation of compound **4** refers to our previous report method [37]. To a solution of **4** (11.2 g, 26 mmol, 1 eq) in MeOH (85 mL) was added PhI(OAc)_2_ (8.8 g, 27.3 mmol, 1.05 eq) at -20 °C. Then, 50% H_2_SO_4_ in MeOH (85 mL) was added by drops. The mixture was stirred for 30 min. The reaction was then quenched with saturated aqueous NaHCO_3_, and diluted by H_2_O (100 mL). The resulting mixture was extracted with EtOAc (3 × 100 mL). The combined organic layers were dried over anhydrous Mg_2_SO_4_, filtered and concentrated under reduced pressure. The residue was purified by flash column chromatography on silica gel with petroleum ether/EtOAc (15:1) to give **5a** and **6a**. 

**5a** or **6a** with the TBS protective group was dissolved in THF (2 mL), and treated with 1.0 M TBAF (1.1 eq) in THF. The mixture was stirred for 0.5 h and diluted by H_2_O (10 mL), and extracted with EtOAc (3 × 10 mL). The combined organic layers were dried over anhydrous Na_2_SO_4_, filtered and concentrated under reduced pressure. The residue was purified by flash column chromatography on silica gel with petroleum ether/EtOAc (4:1) to give compounds **5b** or **6b**, respectively. The mixture **6a** was separated by preparative HPLC to afford **6b-(*Z*)** and **6b-(*E*)** with ratio of 1:1. **6b-(*Z*)**: t_R_ = 18.6 min, purity = 90.4%; **6b-(*E*)**: t_R_ = 20.6 min, purity = 91.8% at 220 nm, 30 min gradient, 40% MeOH: 60% H_2_O to 100% MeOH.

**2-(3-((tert-butyldimethylsilyl)oxy)-4-methoxyphenyl)-1-(3,4-dimethoxyphenyl)ethan-1-one (5a):** yellow viscosity liquid, 61% yield. ^1^H NMR (500 MHz, CDCl_3_): *δ* 7.28 (s, 2H), 6.83 (s, 2H), 6.79 (d, *J* = 1.0 Hz, 1H), 4.16 (s, 2H), 3.93 (s, 3H), 3.91 (s, 6H), 3.81 (s, 3H), 1.00 (s, 9H), 0.15 (s, 6H); ^13^C NMR (125 MHz, CDCl_3_): *δ* 196.77, 153.00 (2C), 149.96, 145.15, 142.47, 131.79, 127.35, 122.30, 122.01, 112.33, 106.31 (2C), 60.93, 56.25 (2C), 55.52, 45.04, 25.72 (3C), 18.45, -4.62 (2C).

**2-(3-hydroxy-4-methoxyphenyl)-1-(3,4,5-trimethoxyphenyl)ethan-1-one (5b):** yellow solid, 95% yield. ^1^H NMR (500 MHz, CDCl_3_): *δ* 7.24 (s, 2H), 6.83 (d, *J* = 2.1 Hz, 1H), 6.76 (d, *J* = 8.3 Hz, 1H), 6.71 (dd, *J* = 8.3, 2.1 Hz, 1H), 5.67 (s, 1H), 4.50–4.46 (m, 1H), 3.85 (s, 3H), 3.84 (s, 6H), 3.82 (s, 3H), 2.40–2.31 (m, 5H), 2.08 – 2.02 (m, 1H), 1.01 (t, *J* = 7.3 Hz, 3H); ^13^C NMR (126 MHz, CDCl_3_): *δ* 211.39, 198.28, 152.90 (2C), 146.08, 145.82, 142.35, 132.55, 131.74, 119.70, 114.42, 111.10, 106.43 (2C), 60.86, 56.22 (2C), 55.93, 51.66, 39.53, 35.96, 27.64, 7.86; MS (ESI): *m/z* calcd for C_18_H_21_O_6_^+^ [M+H] ^+^ 333.13, found 333.34.

**(*Z*)-2-methoxy-5-(2-methoxy-1-(3,4,5-trimethoxyphenyl)vinyl)phenol (6b-(*Z*)):** a yellow solid. ^1^H NMR (500 MHz, CDCl_3_): *δ* 6.76 (d, *J* = 2.1 Hz, 1H), 6.71 (d, *J* = 8.3 Hz, 1H), 6.64 (d, *J* = 2.1 Hz, 1H), 6.54 (s, 2H), 6.32 (s, 1H), 3.82 (s, 3H), 3.79 (s, 3H), 3.72 (s, 6H), 3.68 (s, 3H); ^13^C NMR (126 MHz, CDCl_3_): *δ* 152.74 (2C), 145.49, 145.35, 140.12, 136.79, 133.73, 133.16, 120.14, 119.89, 114.39, 110.48, 107.28 (2C), 60.85, 56.10 (2C), 56.02, 50.89; MS (ESI): *m/z* calcd for C_19_H_23_O_6_^+^ [M+H]^+^ 347.15, found 347.05. 

**(*E*)-2-methoxy-5-(2-methoxy-1-(3,4,5-trimethoxyphenyl)vinyl)phenol (6b-(*E*)):** a yellow solid. ^1^H NMR (500 MHz, CDCl_3_): *δ* 7.06 (d, *J* = 2.1 Hz, 1H), 6.90 − 6.86 (m, 1H), 6.81 (d, *J* = 8.4 Hz, 1H), 6.43 (s, 2H), 6.35 (s, 1H), 3.89 (s, 3H), 3.85 (s, 3H), 3.81 (s, 6H), 3.76 (s, 3H); MS (ESI): *m/z* calcd for C_19_H_23_O_6_^+^ [M+H] ^+^ 347.15, found 347.06. 

#### 3.1.3. Synthesis of *Cis*-Locked Dihydrofuran Analogues **7a**, **7b** and α-Substituted Diphenylethanones **8a**, **8b**

To a solution of compound **5a** (1.0 eq) and Cu(OAc)_2_ (3.0 eq) in DCE was added olefin (3.0 eq), then the mixture was heated to 110 °C, and stirred for 4 h. The reaction was filtered by diatomite, and diluted by H_2_O. The resulting mixture was extracted with EtOAc. The combined organic layers were dried over anhydrous Na_2_SO_4_, filtered and concentrated under reduced pressure. The residue was purified by flash column chromatography on silica gel with petroleum ether/EtOAc to give target compounds with the TBS protective group. Next, compound with TBS group was dissolved in THF (2 mL), and treated with 1.0 M TBAF (1.1 eq) in THF. The mixture was stirred for 0.5 h. The reaction was diluted by H_2_O (10 mL), and extracted with EtOAc (3 × 10 mL). The combined organic layers were dried over anhydrous Na_2_SO_4_, filtered, and concentrated under reduced pressure. The residue was purified by flash column chromatography on silica gel with petroleum ether/EtOAc (4:1) to give **7a** and **7b**.

**2-methoxy-5-(5-phenyl-2-(3,4,5-trimethoxyphenyl)-4,5-dihydrofuran-3-yl)phenol (7a):** yellow solid. Two steps yield: 65%.^1^H NMR (500 MHz, CDCl_3_): *δ* 7.48 (d, *J* = 7.7 Hz, 2H), 7.39 (t, *J* = 7.5 Hz, 2H), 7.32 (t, *J* = 7.0 Hz, 1H), 6.90 (s, 1H), 6.83 (s, 2H), 6.76 (q, *J* = 8.4 Hz, 2H), 5.67 (t, *J* = 9.5 Hz, 1H), 5.54 (s, 1H), 3.86 (s, 3H), 3.86 (s, 3H), 3.72 (s, 6H), 3.54 (dd, *J* = 15.0, 10.4 Hz, 1H), 3.17 (dd, *J* = 15.0, 8.6 Hz, 1H); ^13^C NMR (125 MHz, CDCl_3_): *δ* 152.89 (2C), 148.74, 145.44, 145.13, 143.02, 128.62 (2C), 127.78, 126.91, 125.79 (2C), 119.65, 114.01, 110.58, 108.88, 105.40 (2C), 80.42, 60.91, 56.04 (3C), 44.85; MS (ESI): *m/z* calcd for NaC_26_H_26_O_6_ [M+Na]^+^ 457.16, found 457.49.

**2-methoxy-5-(5-methyl-2-(3,4,5-trimethoxyphenyl)-5-vinyl-4,5-dihydrofuran-3-yl)phenol (7b):** yellow solid. Two steps yield: 45%. ^1^H NMR (500 MHz, CDCl_3_): *δ* 6.85 (s, 1H), 6.76 (s, 2H), 6.73 (s, 2H), 6.11 (dd, *J* = 17.3, 10.7 Hz, 1H), 5.52 (s, 1H), 5.34 (dd, *J* = 17.3, 1.1 Hz, 1H), 5.12 (dd, *J* = 10.7, 1.1 Hz, 1H), 3.86 (s, 3H), 3.84 (s, 3H), 3.72 (s, 6H), 3.09 (d, *J* = 14.9 Hz, 1H), 2.98 (d, *J* = 14.9 Hz, 1H), 1.58 (s, 3H); ^13^C NMR (125 MHz, CDCl_3_): *δ* 152.87 (2C), 147.82, 145.36, 144.94, 142.49, 138.31, 129.50, 127.40, 119.46, 113.83, 112.23, 110.53, 108.37, 105.36 (2C), 83.41, 60.90, 56.04, 56.03 (2C), 48.00, 26.27; MS (ESI): *m/z* calcd for NaC_23_H_26_O_6_ [M+Na]^+^ 421.16, found 421.67.

**2-(3-((*tert*-butyldimethylsilyl)oxy)-4-methoxyphenyl)-1-(3,4,5-trimethoxyphenyl)heptane-1,5-dione (8a):** yellow viscosity liquid, 36% yield. ^1^H NMR (500 MHz, CDCl_3_): *δ* 7.22 (s, 2H), 6.77 (s, 2H), 6.71 (s, 1H), 4.46 (s, 1H), 3.85 (s, 3H), 3.84 (s, 6H), 3.75 (s, 3H), 2.40–2.32 (m, 5H), 2.04 (d, *J* = 7.2 Hz, 1H), 1.02 (s, 3H), 0.95 (s, 9H), 0.09 (s, 6H); ^13^C NMR (125 MHz, CDCl_3_ ): *δ* 211.40, 198.48, 152.89 (2C), 150.14, 145.39, 142.28, 131.90, 131.87, 121.33, 120.91, 112.47, 106.41 (2C), 60.87, 56.21 (2C), 55.46, 51.56, 39.50, 35.96, 27.61, 25.69 (3C), 18.44, 7.87, -4.460, -4.61; MS (ESI): *m/z* calcd for SiC_29_H_43_O_7_ [M+H]^+^ 531.28, found 531.62.

**2-(3-hydroxy-4-methoxyphenyl)-1-(3,4,5-trimethoxyphenyl)heptane-1,5-dione (8b):** white solid, 95% yield. ^1^H NMR (500 MHz, CDCl_3_): *δ* 7.24 (s, 2H), 6.83 (d, *J* = 2.1 Hz, 1H), 6.76 (d, *J* = 8.3 Hz, 1H), 6.71 (dd, *J* = 8.3, 2.1 Hz, 1H), 5.67 (s, 1H), 4.50–4.46 (m, 1H), 3.85 (s, 3H), 3.84 (s, 6H), 3.82 (s, 3H), 2.40–2.31 (m, 5H), 2.08–2.02 (m, 1H), 1.01 (t, *J* = 7.3 Hz, 3H); ^13^C NMR (125 MHz, CDCl_3_): *δ* 211.39, 198.28, 152.90 (2C), 146.08, 145.82, 142.35, 132.55, 131.74, 119.70, 114.42, 111.10, 106.43 (2C), 60.86, 56.22 (2C), 55.93, 51.66, 39.53, 35.96, 27.64, 7.86; MS (ESI): *m/z* calcd for NaC_23_H_28_O_7_ [M+Na]^+^ 439.17, found 439.46.

#### 3.1.4. Synthesis of Cyclobutane Analogue **9**

To a solution of **8a** (0.5 mmol, 1.0 eq) in dry THF (10 mL) was added 1 M LiHMDS solution in THF (1.0 mL, 1.0 mmol, 2.0 eq) at −78 °C under argon atmosphere. After stirred for 1 h, the reaction was added *p*-toluensulfonyl chloride (TsCl, 191 mg, 1.88 mmol, 2.0 eq) solution in THF (2 mL). The reaction stirred overnight, and quenched with saturated aqueous NH_4_Cl, diluted by H_2_O (10 mL). The resulting mixture was extracted with EtOAc (3 × 10 mL). The combined organic layers were dried over anhydrous Mg_2_SO_4_, filtered, and concentrated under reduced pressure. The flash column chromatography on silica gel with petroleum ether/EtOAc (8:1) gave **9**.

**1-(3-(3-((*tert*-butyldimethylsilyl)oxy)-4-methoxyphenyl)-2-hydroxy-2-(3,4,5-trimethoxyphenyl)cyclobutyl)propan-1-one (9):** yellow solid, 40% yield. ^1^H NMR (500 MHz, CDCl_3_): *δ* 6.70 (d, *J* = 0.8 Hz, 2H),6.63 (s, 1H), 6.45 (s, 2H), 3.80 (s, 3H), 3.78 (s, 6H), 3.73 (s, 3H), 3.69 (d, *J* = 3.4 Hz, 1H), 3.50 (dd, *J* = 12.1, 3.3 Hz, 1H), 2.72–2.52 (m, 4H), 2.10 (s, 1H), 0.92 (s, 12H), 0.03 (s, 6H); ^13^C NMR (126 MHz, CDCl_3_): *δ* 213.47, 152.69 (2C), 150.01, 144.79, 139.75, 136.98, 132.28, 122.09, 122.02, 111.97, 103.82 (2C), 80.85, 0.81, 56.89, 56.17 (2C), 55.42, 47.27, 38.30, 27.28, 25.66 (3C), 18.36, 12.99, −4.66, −4.72; MS (ESI): *m/z* calcd for SiC_29_H_43_O_7_ [M+H]^+^ 531.28, found 531.74.

#### 3.1.5. Synthesis of Cyclohexane Analogue **10a**, **10b** and **11**, **12**

To a solution of **8a** (1.87 g, 35.2 mmol, 1.0 eq) in MeOH (10 mL) was added LiOH (844 mg, 35.2 mmol, 1.0 eq) at 0 °C. After stirring for 0.5 h, the solution was quenched with saturated aqueous NH_4_Cl, diluted by H_2_O (20 mL). The resulting mixture was extracted with EtOAc (3 × 20 mL). The combined organic layers were dried over anhydrous Na_2_SO_4_, filtered, and concentrated under reduced pressure. The residue was purified by flash column chromatography on silica gel with petroleum ether/EtOAc (10: 1) to give 560 mg compounds **10a**. 

To a solution of **10a** (20 mg, 0.38 mmol, 1.0 eq) in THF (2 mL) was added 1.0 M TBAF (42 µL, 0.42 mmol, 1.1 eq) in THF. The mixture was stirred for 0.5 h. The reaction was diluted by H_2_O (10 mL), and extracted with EtOAc (3 × 10 mL). The combined organic layers were dried over anhydrous Na_2_SO_4_, filtered and concentrated under reduced pressure. The residue was purified by flash column chromatography on silica gel with petroleum ether/EtOAc (4:1) to give 15 mg compounds **10b**.

To a solution of **10a** (30 mg, 56 µmol, 1.0 eq) in dry DCM (5 mL) was added NaBH_4_ (7.6 mg, 113 µmol, 2.0 eq) at 0 °C. After stirred for 2 h, the reaction was then quenched with saturated aqueous NH_4_Cl, and diluted by H_2_O (5 mL). The resulting mixture was extracted with EtOAc (5 × 10 mL). The combined organic layers were dried over anhydrous Mg_2_SO_4_, filtered and concentrated under reduced pressure. The residue was redissolved in THF (3 mL), and treated with 1.0 M TBAF (62 µL, 62 µmol, 1.1 eq) in THF. The mixture was stirred for 0.5 h. The reaction was diluted by H_2_O (5 mL), and extracted with EtOAc (3 × 5 mL). The combined organic layers were dried over anhydrous Na_2_SO_4_, filtered and concentrated under reduced pressure. The residue was purified by flash column chromatography on silica gel with petroleum ether/EtOAc (2: 1) to give 10 mg compounds **11**.

To a solution of **10a** (500 mg, 0.94 mmol, 1.0 eq) in dry THF (10 mL) was added 1 M LiHMDS solution in THF (1.88 mL, 1.88 mmol, 2.0 eq) at -78 °C under argon atmosphere. After being stirred for 1 h, the reaction was added to TsCl (360 mg, 1.88 mmol, 2.0 eq) solution in THF (3 mL). The reaction stirred overnight, and quenched with saturated aqueous NH_4_Cl, diluted by H_2_O (10 mL). The resulting mixture was extracted with EtOAc (3 × 10 mL). The combined organic layers were dried over anhydrous Mg_2_SO_4_, filtered and concentrated under reduced pressure. The residue was redissolved in DMF (3 mL), and added DMAP (12 mg, 94 µmol, 0.1 eq) and Et_3_N (1.3 mL, 9.4 mmol, 10 eq). The reaction was heated to 80 °C, and stirred overnight, then quenched with saturated aqueous NH_4_Cl, diluted by H_2_O (30 mL). The resulting mixture was extracted with EtOAc (3 × 20 mL). The combined organic layers were dried over anhydrous Na_2_SO_4_, filtered and concentrated under reduced pressure. The residue was redissolved in THF (5 mL), and treated with 1.0 M TBAF (940 µL, 0.94 mmol, 1.0 eq) in THF. The mixture was stirred for 0.5 h. The reaction was diluted by H_2_O (10 mL), and extracted with EtOAc (3 × 10 mL). The combined organic layers were dried over anhydrous Na_2_SO_4_, filtered, and concentrated under reduced pressure. The residue was purified by flash column chromatography on silica gel with petroleum ether/EtOAc (4:1) to give 93 mg compounds **12** as a white solid.

**4-(3-((*tert*-butyldimethylsilyl)oxy)-4-methoxyphenyl)-3-hydroxy-2-methyl-3-(3,4,5-trimethoxyphenyl)cyclohexan-1-one (10a):** white solid, 65% yield. ^1^H NMR (500 MHz, CDCl_3_ ): *δ* 6.56 (d, *J* = 7.9 Hz, 1H), 6.48 (d, *J* = 8.3 Hz, 2H), 3.77 (s, 3H), 3.68 (s, 3H), 3.35 (d, *J* = 11.2 Hz, 1H), 3.08 (d, *J* = 6.5 Hz, 1H), 2.72 – 2.52 (m, 3H), 2.09 (d, *J* = 11.8 Hz, 1H), 1.97 (s, 1H), 0.92 (s, 9H), 0.80 (d, *J* = 6.3 Hz, 3H), 0.02 (s, 3H), -0.02 (s, 3H); ^13^C NMR (125 MHz, CDCl_3_ ): *δ* 210.40, 149.77, 144.33, 140.22, 136.61, 132.41, 121.77, 121.76, 111.50, 82.95, 60.84, 56.18, 56.16, 55.48, 53.62, 52.88, 41.56, 28.97, 25.67 (3C), 18.36, 8.15, -4.75, -4.84; MS (ESI): *m/z* calcd for SiC_29_H_43_O_7_ [M+H]^+^ 531.28, found 531.63.

**3-hydroxy-4-(3-hydroxy-4-methoxyphenyl)-2-methyl-3-(3,4,5-trimethoxyphenyl)cyclohexan-1-one (10b):** white solid, 95% yield. ^1^H NMR (500 MHz, DMSO-D6): *δ* 8.49 (s, 1H), 6.57 (d, *J* = 1.5 Hz, 1H), 6.55 (d, *J* = 8.3 Hz, 1H), 6.35 (d, *J* = 8.2 Hz, 1H), 4.94 (s, 1H), 3.62 (s, 3H), 3.56 (s, 3H), 3.39 (dd, *J* = 12.6, 3.3 Hz, 1H), 3.34 - 3.31 (m, 1H), 2.71 (td, *J* = 13.3, 6.6 Hz, 1H), 2.46 − 2.31 (m, 2H), 1.90–1.83 (m, 1H), 0.66 ppm (d, *J* = 6.7 Hz, 3H); ^13^C NMR (125 MHz, DMSO-D6 ): *δ* 211.12, 146.07, 145.68, 141.49, 135.85, 135.35, 120.18, 116.97, 111.59, 82.75, 60.42, 55.96, 53.75, 52.64, 41.40, 29.85, 8.79; MS (ESI): *m/z* calcd for NaC_23_H_28_O_7_ [M+Na]^+^ 439.17, found 439.59.

**6-(3-hydroxy-4-methoxyphenyl)-2-methyl-1-(3,4,5-trimethoxyphenyl)cyclohexane-1,3-diol (11):** white solid, two steps yield: 66%. ^1^H NMR (500 MHz, CDCl_3_): *δ* 7.66 (dd, *J* = 259.2, 6.5 Hz, 1H), 6.52 (s, 1H), 6.50 (s, 2H), 6.20 (d, *J* = 6.9 Hz, 1H), 6.11 (s, 1H), 5.48 (s, 1H), 3.90 (s, 3H), 3.84 (d, *J* = 10.0 Hz, 1H), 3.79 (s, 3H), 3.76 (s, 3H), 3.54 (s, 3H), 2.86 (d, *J* = 12.1 Hz, 1H), 2.48 (s, 1H), 2.24 (dd, *J* = 27.5, 12.3 Hz, 2H), 1.98 (s, 1H), 1.63 (dd, *J* = 23.6, 11.6 Hz, 2H), 0.86 (s, 3H); ^13^C NMR (125 MHz, CDCl_3_): *δ* 145.10, 144.96, 141.62, 136.48, 134.03, 130.25, 127.08, 120.49, 114.73, 110.00, 103.80, 101.65, 79.72, 72.37, 60.92, 56.38, 56.09, 55.86, 53.40, 48.04, 35.44, 26.56, 11.48; MS (ESI): *m/z* calcd for NaC_23_H_30_O_7_ [M+Na]^+^ 441.19, found 441.68.

**5-(3-hydroxy-4-methoxyphenyl)-1-methyl-6-(3,4,5-trimethoxyphenyl)-7-oxabicyclo[4.1.0]heptan-2-one (12):** white solid, three steps yield: 28%. ^1^H NMR (500 MHz, CDCl_3_): *δ* 6.80 (s, 1H), 6.60 (t, *J* = 5.6 Hz, 2H), 6.32 (s, 2H), 5.48 (s, 1H), 3.79 (s, 3H), 3.76 (s, 3H), 3.43 (d, *J* = 10.9 Hz, 1H), 2.73 (d, *J* = 15.3 Hz, 1H), 2.44 – 2.29 (m, 2H), 1.91 – 1.84 (m, 1H), 1.13 (s, 3H); ^13^C NMR (125 MHz, CDCl_3_): *δ* 205.58, 152.78 (2C), 145.34, 145.26, 137.09,134.93, 132.67, 119.84, 114.35, 110.31, 72.21, 65.16, 60.82, 56.19, 55.84 (2C), 45.67, 37.13, 26.33, 13.05; MS (ESI): *m/z* calcd for NaC_23_H_26_O_7_ [M+Na]^+^ 437.16, found 437.34.

### 3.2. Biological Activity Evaluation

#### 3.2.1. Cell Culture

The HepG2 cell line was originally acquired from Shanghai Institute of Biochemistry and Cell Biology, Chinese Academy of Sciences. This cell line was grown in RPMI-1640 (Gibco) including 10% (v/v) thermally inactivated fetal bovine serum (FBS), penicillin (100 KU/L) and streptomycin (100 KU/L) at 37 °C in a 5% CO_2_ humidified incubator.

#### 3.2.2. Cytotoxic Activity (SRB Assay)

In vitro cytotoxic activity was evaluated by using the SRB colorimetric assay as previous methods [38,41]. HepG2 cells were treated with analogues for 48 h at various concentrations. 

#### 3.2.3. Apoptosis-Inducing Activity (PI/Annexin V-FITC Assay)

The apoptosis in HepG2 cells was detected with a propidium iodide (PI)/Annexin V-FITC apoptosis detection kit (KeyGEN Biotech, Nanjing, China) as described previously [42]. After the treatment of **6b-(*Z*)**, **6b-(*E*)** and CA-4 at the indicated concentrations for 48 h, HepG-2 cells were stained with PI and Annexin V-FITC and then were resuspended in 400 μL of binding buffer. Flow cytometric analysis (FACS Calibur; Becton-Dickinson, San Jose, CA, US) was performed. A total of 10000 cells were acquired per sample and data were analyzed using Cellquest software (Becton-Dickinson, San Jose, CA, US). 

#### 3.2.4. Cell Cycle Distribution (PI Staining Assay)

Cell cycle distribution on HepG2 cells was assessed by flow cytometry. PI staining assay (Sigma) was used for the cell cycle arrest of cells as described previously [40]. After treatment with test compound **1** at the indicated concentrations for 24 h, cells were centrifuged and fixed in 70% ethanol at 4 °C overnight and subsequently resuspended in PBS containing 100 μL RNase A and 400 μL PI. Cellular DNA content, for cell cycle distribution analysis, was measured using a FACS Calibur flow cytometer (Becton-Dickinson, San Jose, CA, US) and analyzed using a CellQuest software package (Becton-Dickinson, San Jose, CA, US). Twenty thousand events were collected per sample. Mean values from 3 independent experiments are presented. The results were analyzed using Modfit LT 3.0 software.

#### 3.2.5. Immunofluorescence Analysis (DAPI /α-Tubulin-TRITC Assay)

Immunofluorescence effects of **6b-(*E*)** and CA-4 on the inhibition of tubulin polymerization in HepG-2 cells were measured as described previously [37]. HepG-2 cells were incubated in 8-well cell slide (Eppendorf AG, Hauppauge, NY). After treatment for 48 h at the indicated concentrations of **6b-(*E*)** (0.3 μM) and CA-4 (0.02 μM), the cells was fixed with 4% paraformaldehyde, blocked with PBS containing 5% BSA and incubated with anti-α-tubulin-TRITC antibodies (1:500, Abcam Trading Company Ltd., Shanghai, China) and the secondary antibody (goat anti-rabbit IgG, 1:500, ZSGB-BIO Inc., Beijing, China). At the last step, the cells were stained with DAPI. Images of cells were acquired using an Olympus fluorescence microscope with a 20× objective.

#### 3.2.6. Tubulin Polymerization Assay

Tubulin polymerization assay were performed in the light of the fluorescence based tubulin polymerization assay kit’s protocol (Cat. #BK011P, Cytoskeleton Inc., Denver, CO). Simplify, tubulin (10 mg/mL) containing buffer 1, tubulin glycerol buffer and GTP stock (100 mM) were incubated with **6b-(*E*)** (5, 20 and 40 µM), paclitaxel (3 µM), CA-4 (3 µM) and DMSO for 50 min at 37 °C. The fluorescence increase was monitored every minute for the characterization of tubulin polymerization at 360 nm excitation wavelength and 460 nm emission wavelengths by microplate spectrophotometer (Synergy HTX, BioTek Instruments, Inc., Winooski, VT). 

#### 3.2.7. Statistics Analysis

All the data were shown as mean ± standard deviation (SD) from at least three separate determinations with three duplicates. Statistical analysis was performed using GraphPad Prism software with the 2-tailed Student *t*-test.

### 3.3. Molecular Docking

All docking analysis was performed by using the Surflex-Dock program in the Sybyl-X 2.0 software (Tripos Inc., St. Louis, MO). The X-ray crystal structure of tubulin protein (PDB ID: 1SA0) was acquired from the RSCB Protein Data Bank (http://www.rcsb.org/pdb). All bound waters and ligands were eliminated from the protein and the polar hydrogen was added to the proteins. Compounds **6b-(*Z*)** and **6b-(*E*)** were drawn and optimized automatically in the Sybyl-X 2.0 software, and saved as a mol2 file. Default parameters were used for Docking. The analysis of results was carried out by USCF Chimera software, which was downloaded from http://www.cgl.ucsf.edu/chimera.

## 4. Conclusions

In this work, we constructed a series of novel CA-4 analogues on the *cis*-double bond containing diphenylethanone, *cis*-locked dihydrofuran, α-substituted diphenylethanone, cyclobutane and cyclohexane. Yet, most compounds exhibited weak cytotoxic activity against HepG2 cells. Unexpectedly, a by-product **6b** (*cis–trans* isomers) in the preparation of diphenylethanone analogues was found to be the most potent cytotoxic agents against HepG2 cells with IC_50_ values of less than 0.5 μM. The two isomers of **6b** both induced cellular apoptosis, significantly arrested cells in the G2/M phase, severely disrupted microtubule network in HepG2 cells. An in vitro tubulin polymerization assay revealed that **6b-(*E*)** was a dose-dependent and effective inhibitor of the tubulin assembly. In addition, molecular docking showed that two stereoisomers of **6b** bound similarly and efficiently at the colchicine binding site on tubulin well with CA-4 in similar distorted conformations. Although the anticancer activity of all novel compounds did not behave as excellently as CA-4, these results provided fundamental data for new developments of tubulin inhibitors. Additional insight on **6b** will expand our research of olefin modification of CA-4 or *iso*CA-4 as tubulin polymerization agents.

## Figures and Tables

**Table 1:** Cytotoxic activities (IC_50_) of compounds against HepG2 cells.

Compd	IC_50_ (µM) ^1^	Compd	IC_50_ (µM)
**5b**	> 10	**9**	> 10
**6b-(*Z*)**	0.30 ± 0.05	**10a**	> 10
**6b-(*E*)**	0.23 ± 0.07	**10b**	> 10
**7a**	> 10	**11**	> 10
**7b**	> 10	**12**	3.67± 0.16
**8a**	> 10	CA-4	0.013 ± 0.003
**8b**	> 10	*iso*CA-4	0.017 ± 0.002

^1^ The IC_50_ values represent the concentration that causes 50% inhibition of cell viability measured by SRB assay. Cells were treated with the compounds for 48 h. All data (mean ± SD) are the average of three determinations. HepG-2: human liver cancer cells.

## Supplementary Materials

Supplementary materials can be found at https://www.mdpi.com/1422-0067/21/5/1817/s1.
