# Supplementary material for: Exploring Diverse-Ring Analogues on Combretastatin A4 (CA-4) Olefin as Microtubule-Targeting Agents
Source: Int J Mol Sci. 2020 Mar 6;21(5):1817. doi: 10.3390/ijms21051817 (PMC7084768; doi:10.3390/ijms21051817)

Supplementary Materials

Exploring diverse-ring analogues on combretastatin A4 (CA-4) olefin as microtubule-targeting agents

Ming-Yu Song [1,†], Qiu-Rui He [1,†], Yi-Lin Wang [2], Hao-Ran Wang [2], Tian-Cheng Jiang [3], Jiang-Jiang Tang [1,*] and Jin-Ming Gao [1]

[1] Shaanxi Key Laboratory of Natural Products & Chemical Biology, College of Chemistry & Pharmacy, Northwest A&F University; mingyusong@nwsuaf.edu.cn (M.Y.S.); heqiurui@nwsuaf.edu.cn (Q.R.H.); jinminggao@nwsuaf.edu.cn (J.M.G.)
[2] College of Innovation and Experiment, Northwest A&F University; 791279959@qq.com (Y.L.W.); 233712828@qq.com (H.R.W.)
[3] College of Food Science and Engeering, Northwest A&F University; 942208204@qq.com (T.C.J.)
† These authors contributed equally to this work.
* Correspondence: tangjiang11@nwsuaf.edu.cn (J.J.T.); Tel.: +86-2987-09-2662 (J.J.T.)

List of Contents:

Page S2. **Scheme S1.** Possible mechanism for formation of **5a** and **6a**.

Page S2. **Figure S1.** Predicted binding mode of CA-4, **6b-(*Z*)** and **6b-(*E*)**.

Pages S3-S17. **Figure S2-S15**. NMR spectra of all analogues.

Pages S18. **Figure S16.** HPLC preparation of **6b-(*Z*)** and **6b-(*E*).**

Scheme S1. Possible mechanism for formation of 5a and 6a by H2SO as an activator.

The intermediate **2** has a better activity than **1** in the case of high electron density, causing a higher yield of **5a** in our work. To the formation of the by-product **6a**, MeOH may attack competitively the intermediate **2** in reaction kinetics to trigger the formation of **6a** through following elimination.

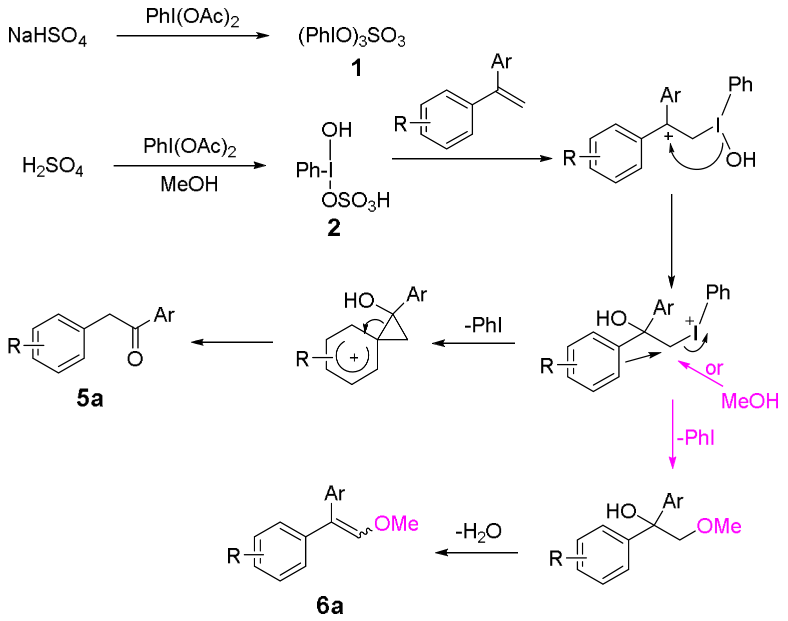

Figure S1. Predicted binding mode of CA-4, **6b-(*Z*)** and **6b-(*E*)** with the colchicines binding site of tubulin (PDB code: 1SA0). Surrounding key amino acid residues are labeled, and hydrogen bonds are shown by yellow dashed lines. (A) CA-4 (blue cyan), (B) **6b-(*Z*)** (magenta) and (c) **6b-(*E*)** (cyan).

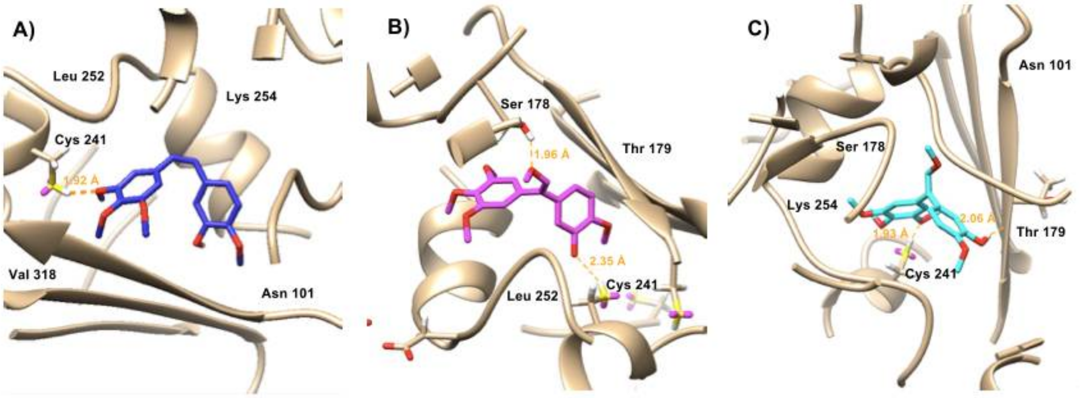

Figure S2. ^{1}H, DEPT135 and ^{13}C NMR ($CDCl_3$) spectra of compound **5a**.

^{1}H NMR (500 MHz, $CDCl_3$)

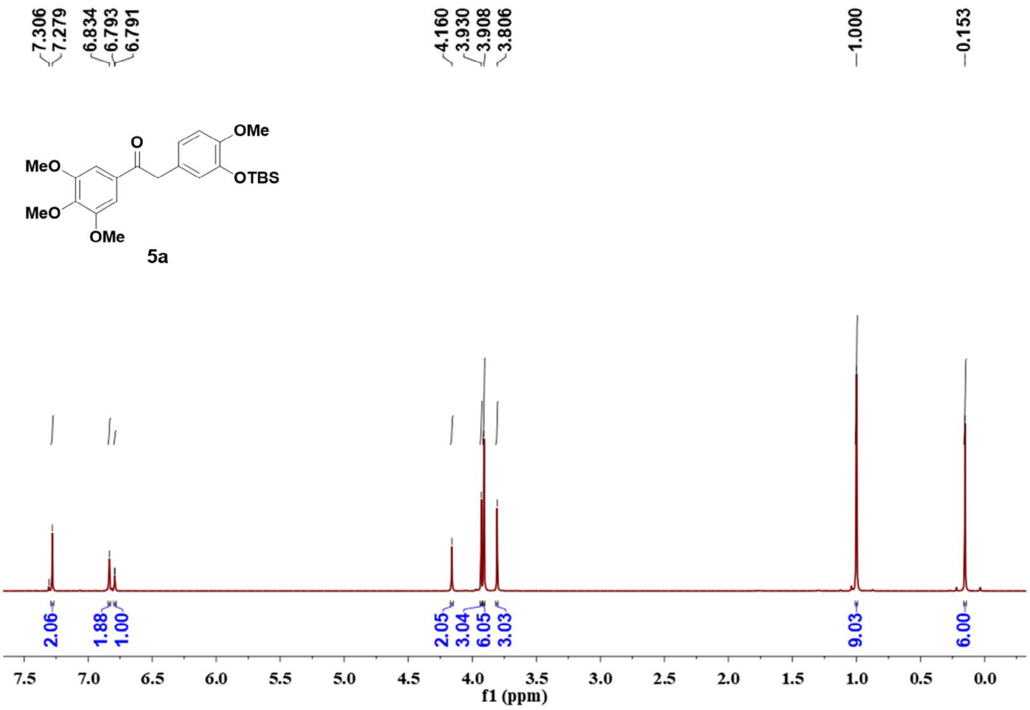

^{13}C NMR (125 MHz, $CDCl_3$)

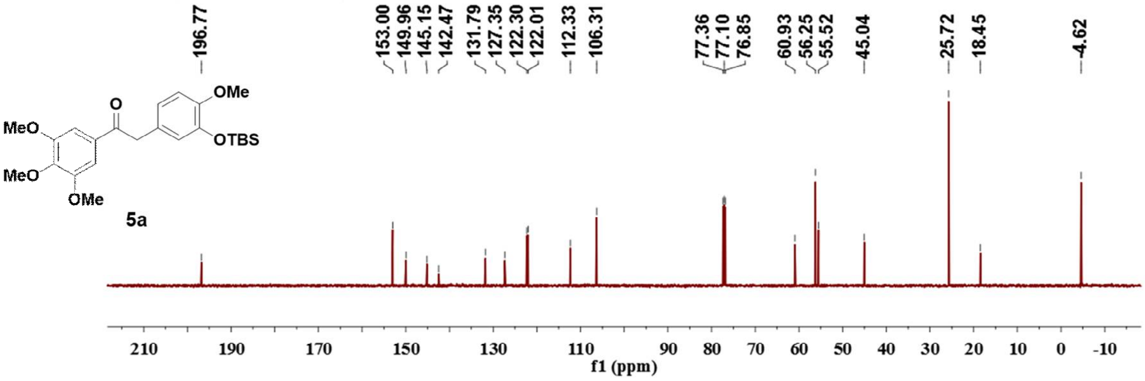

^{13}C DPET135

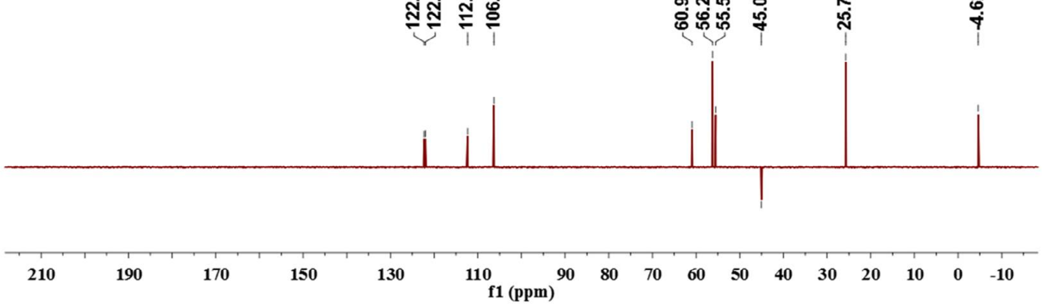

Figure S3. ^{1}H, DEPT135 and ^{13}C NMR (CDCl$_3$) spectra of compound **5b**.

^{1}H NMR (500 MHz, CDCl$_3$)

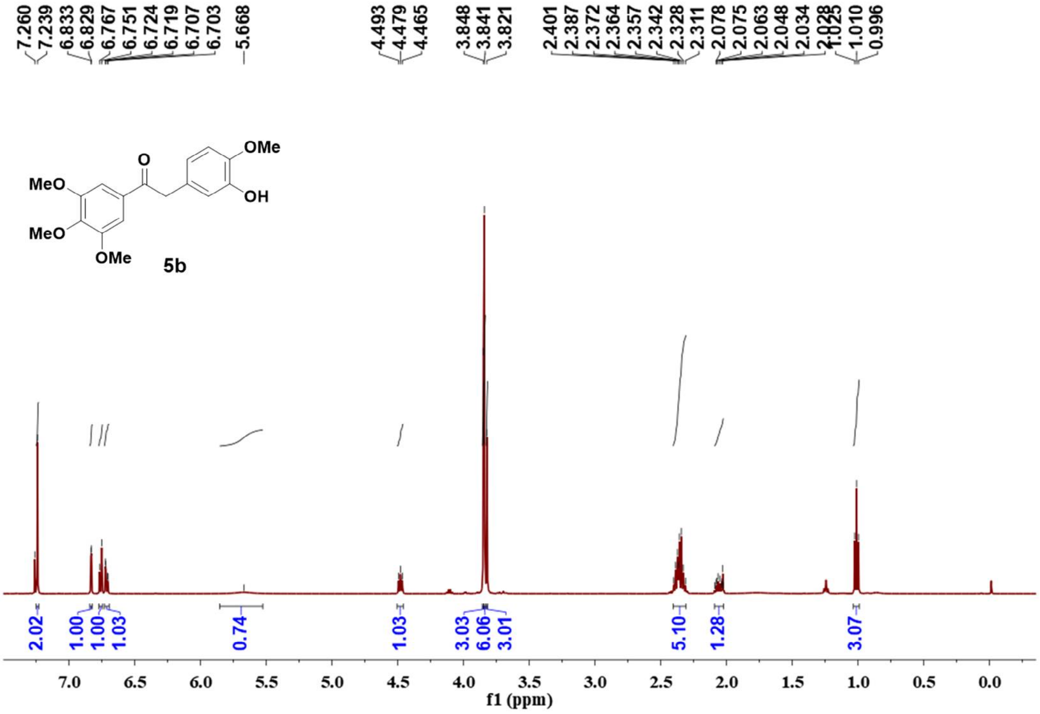

^{13}C NMR (125 MHz, CDCl$_3$)

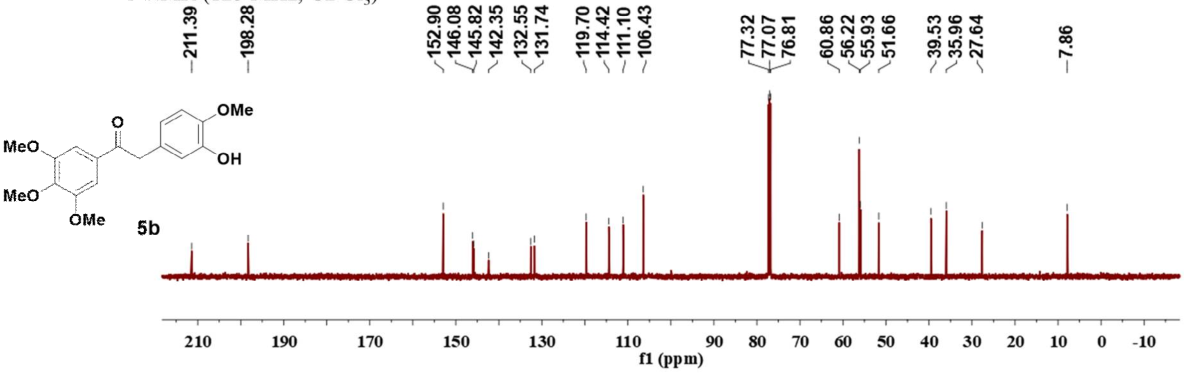

^{13}C DPET135

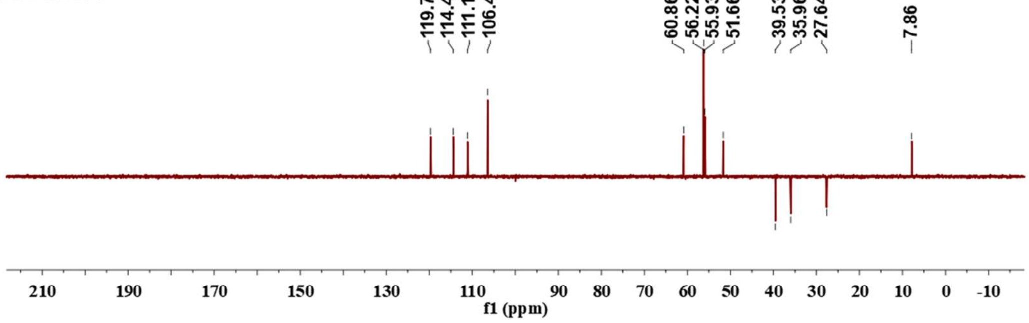

Figure S4. ^{13}C NMR spectra of mixture of **6b-(*Z*)** and **6b-(*E*)**, the ratio is about 1:1.

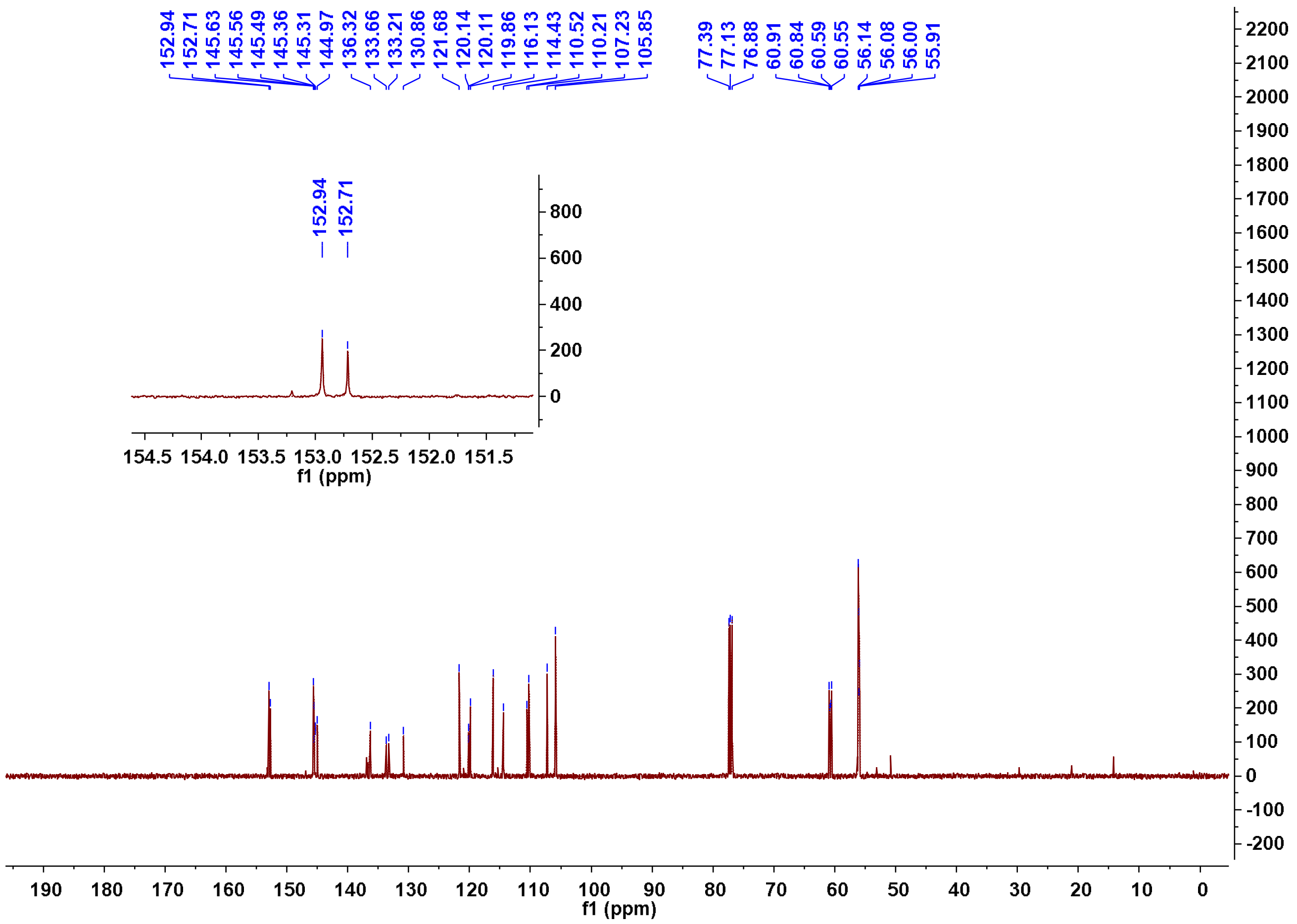

Figure S5. ^{1}H, ^{13}C NMR and 2D NOESY ($CDCl_3$) spectra of compound **6b-(Z)**.

^{1}H NMR (500 MHz, $CDCl_3$)

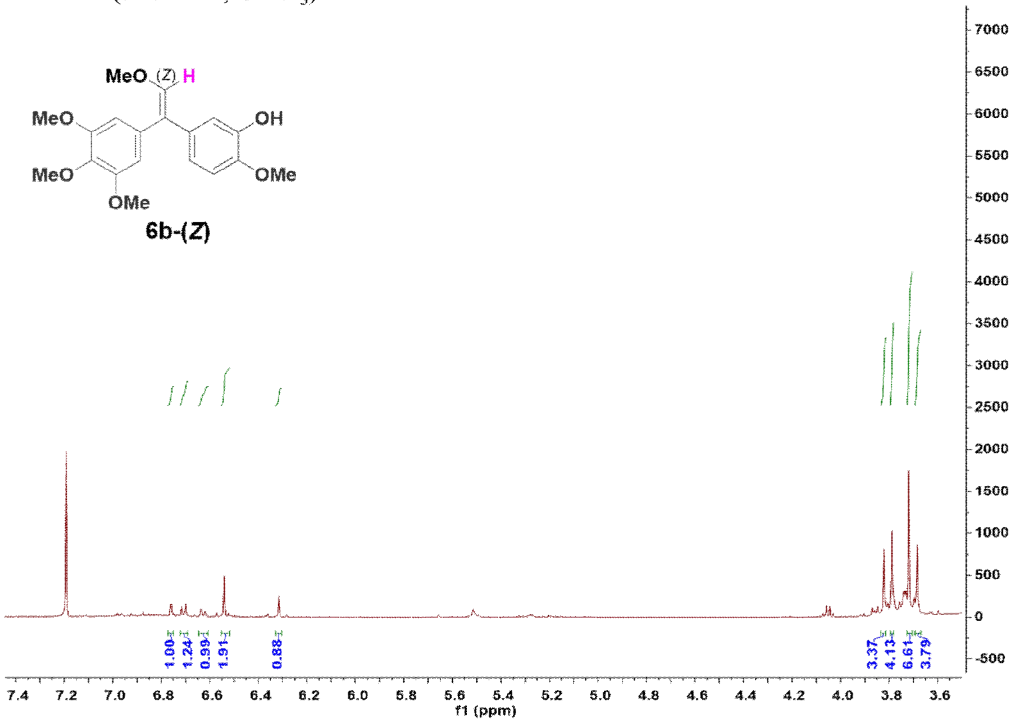

^{13}C NMR (125 MHz, $CDCl_3$)

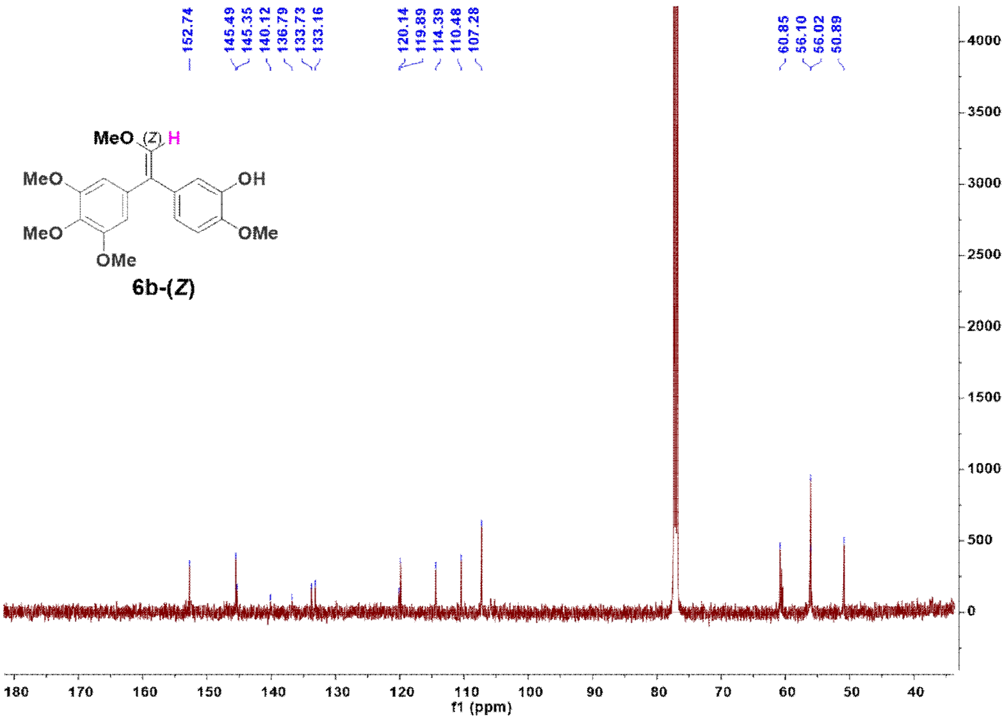

NOESY

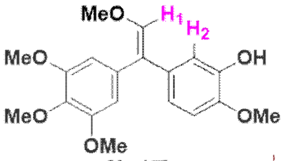

6b-(*Z*)

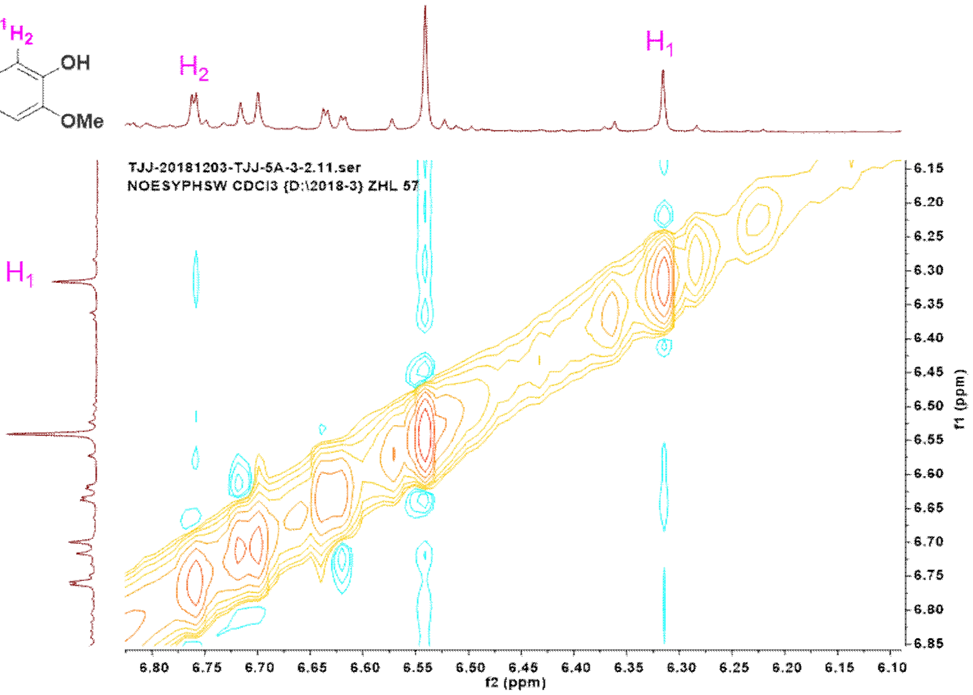

**Parameter of NOESY**	**Value**
Title	TJJ-20181203-TJJ-5A-3-2.11.ser
Origin	Bruker BioSpin GmbH
Owner	Administrator
Solvent	CDCl3
Temperature	300.0
Pulse Sequence	noesyph
Probe	5 mm PABBO BB-1H/ D Z-GRD Z113652/ 0062
Number of Scans	32
Receiver Gain	64.0
Relaxation Delay	1.9980
Pulse Width	12.6000
Acquisition Time	0.2028
Acquisition Date	2018-12-03T21:58:01
Modification Date	2018-12-04T04:27:56
Spectrometer Frequency	(500.13, 500.13)
Spectral Width	(5050.5, 5050.5)
Lowest Frequency	(-743.3, -743.3)
Nucleus	(1H, 1H)
Acquired Size	(1024, 256)
Spectral Size	(1024, 1024)

Figure S6. ^{1}H NMR ($CDCl_3$) spectra of compound **6b-(E)**.

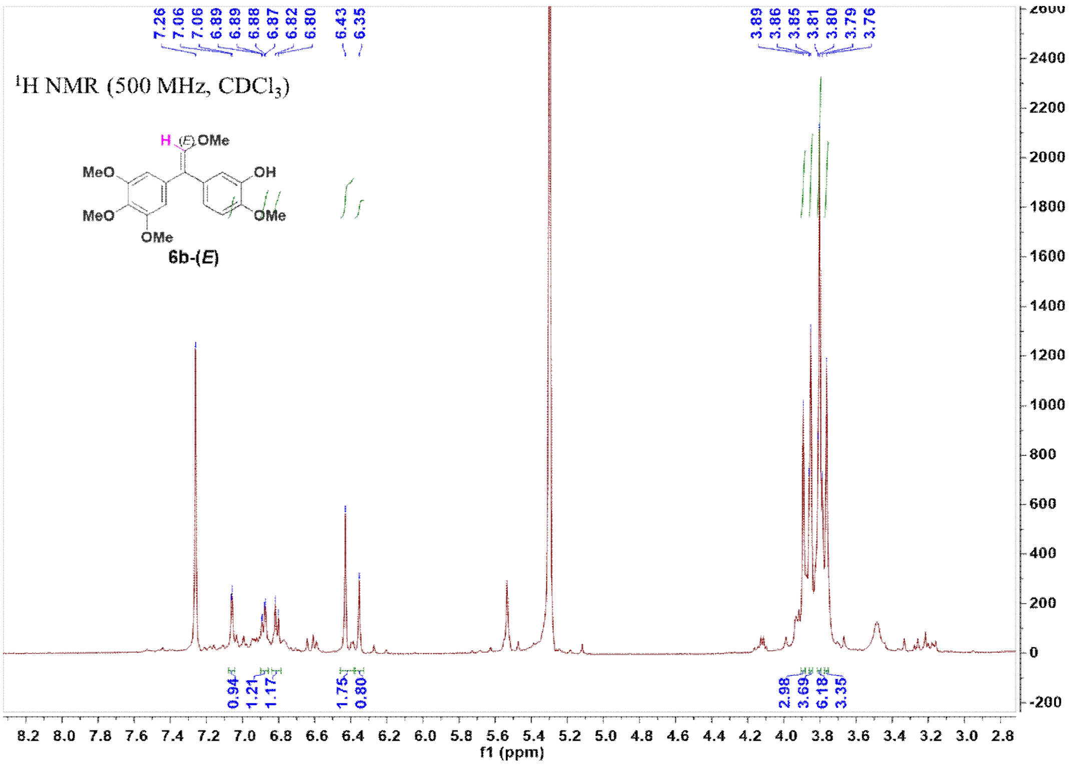

Figure S7. ^{1}H, DEPT135 and ^{13}C NMR ($CDCl_3$) spectra of compound **7a**.

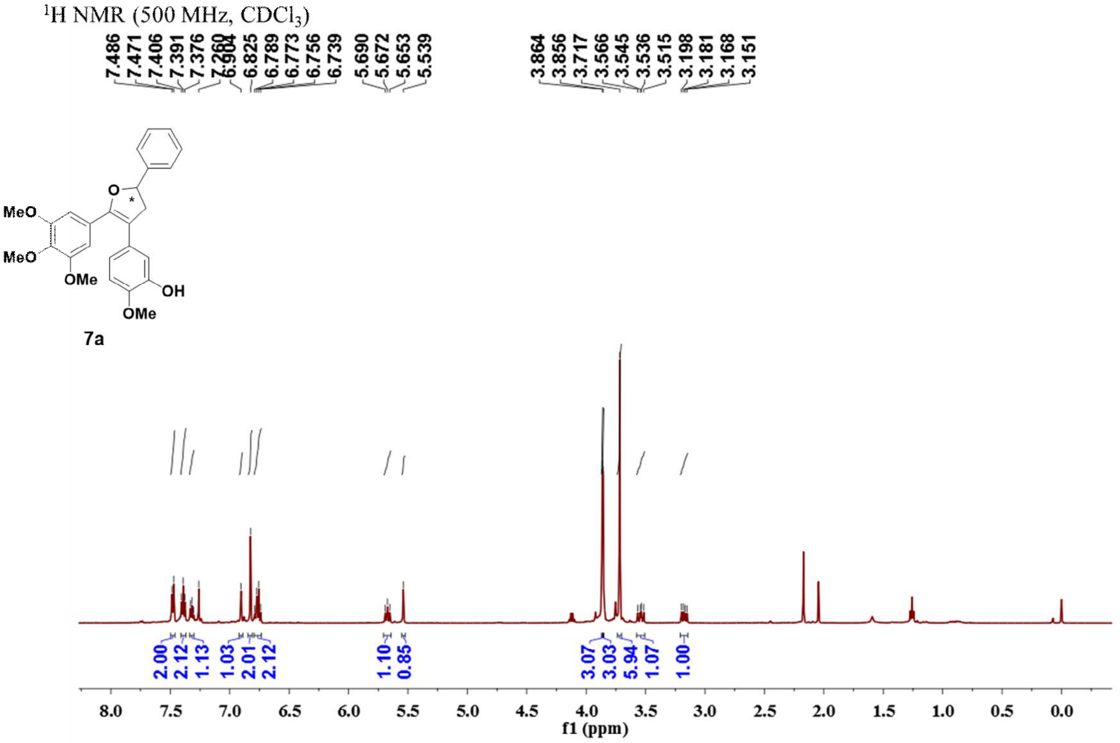

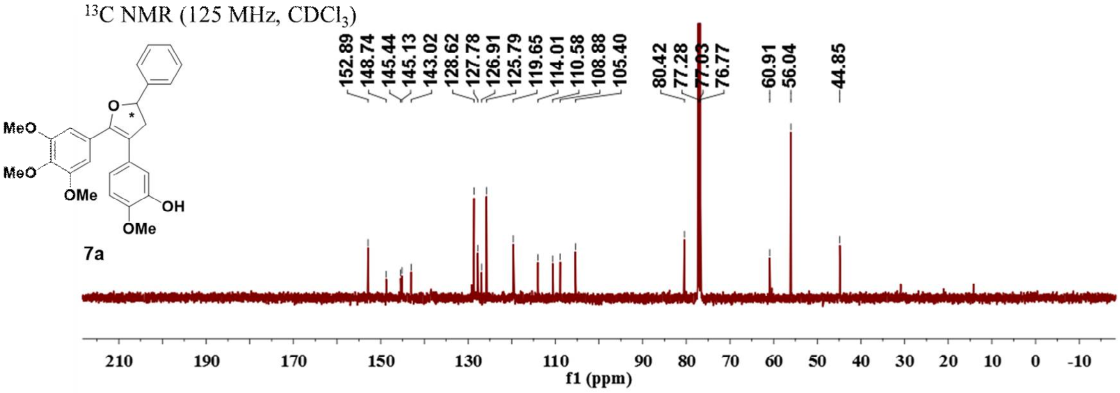

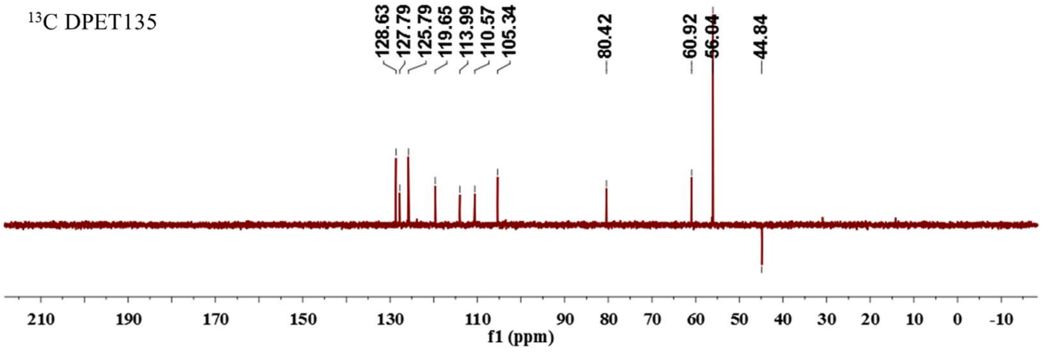

Figure S8. ^{1}H, DEPT135 and ^{13}C NMR ($CDCl_3$) spectra of compound **7b**.

^{1}H NMR (500 MHz, $CDCl_3$)

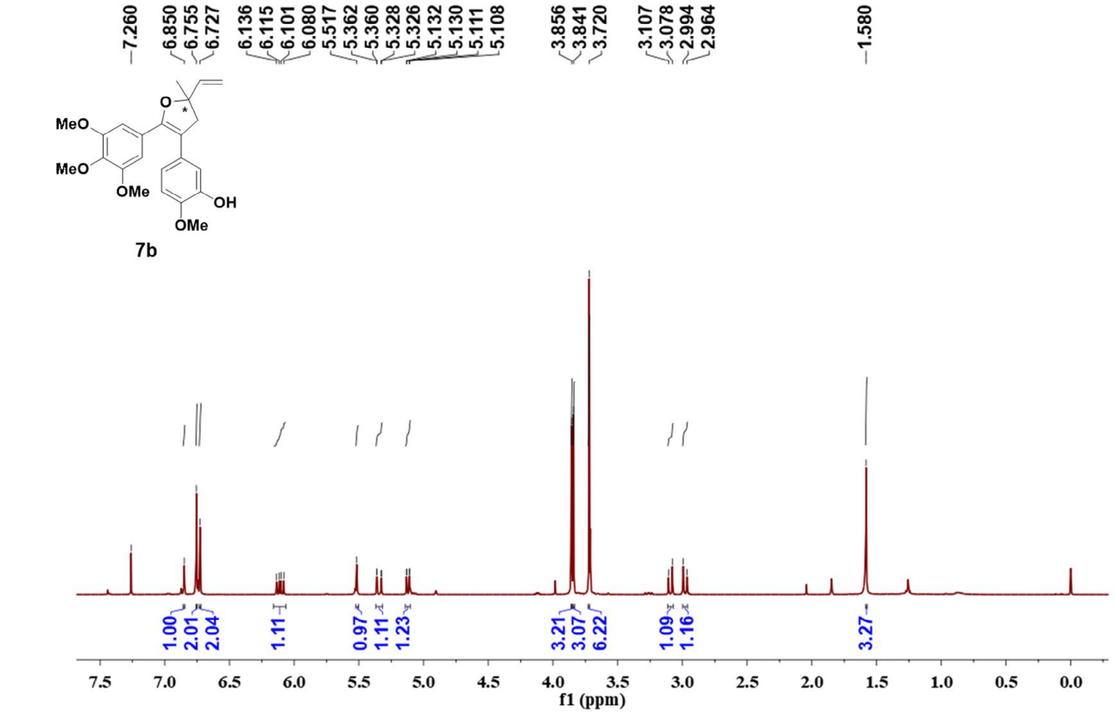

^{13}C NMR (125 MHz, $CDCl_3$)

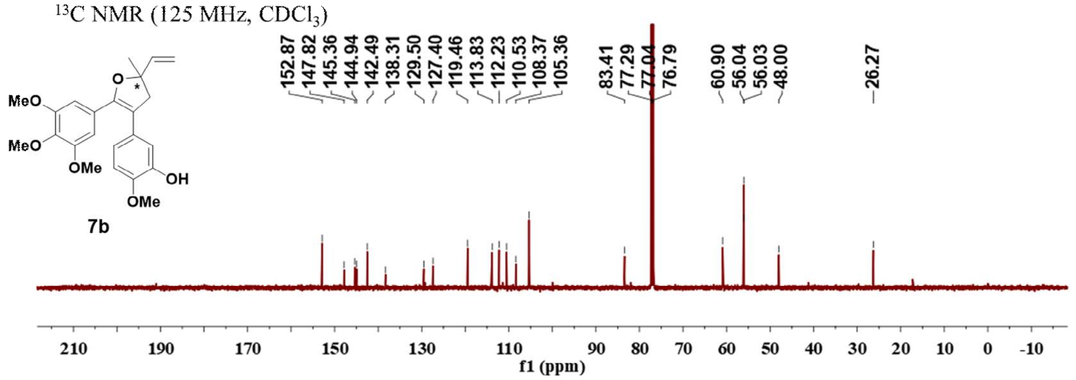

^{13}C DPET135

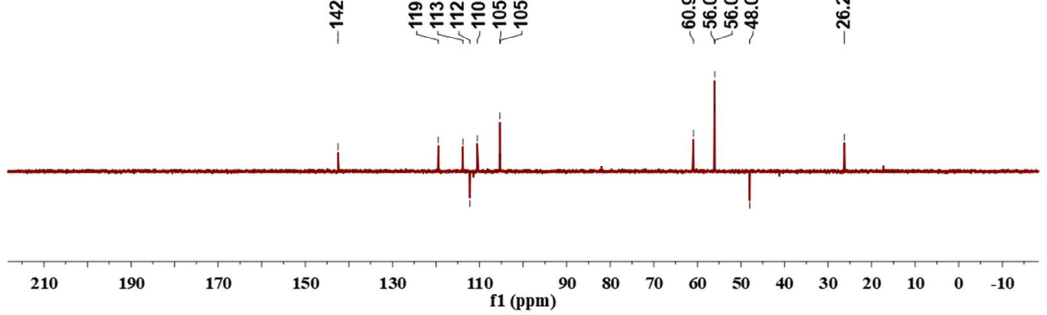

Figure S9. ^{1}H, DEPT135 and ^{13}C NMR (CDCl$_3$) spectra of compound **8a**.

^{1}H NMR (500 MHz, CDCl$_3$)

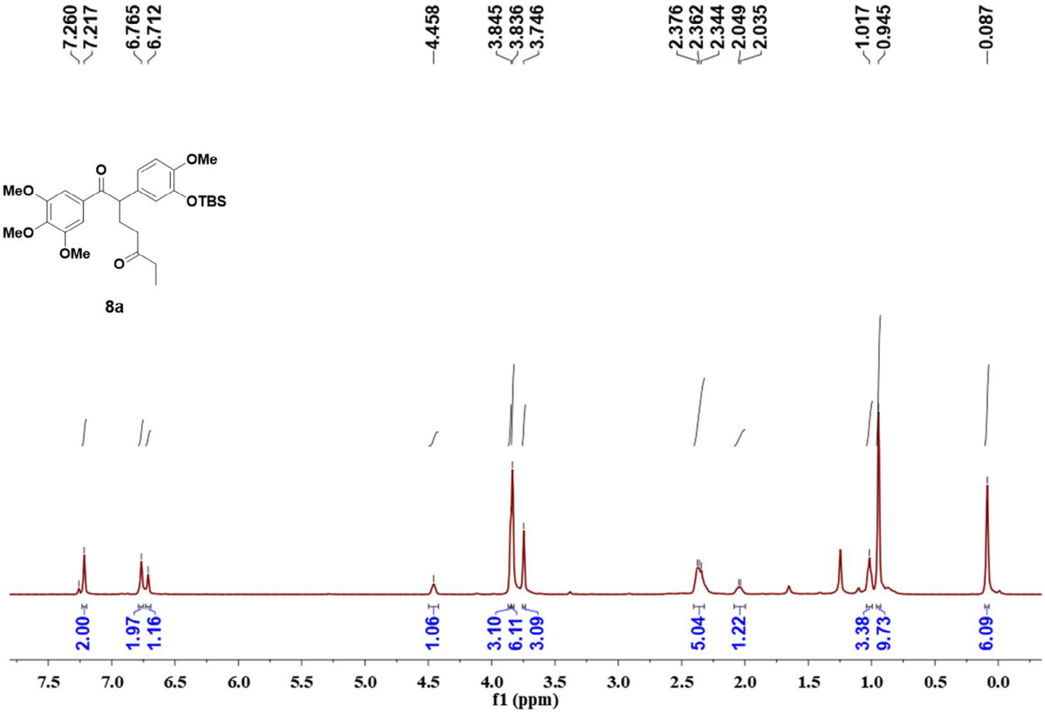

^{13}C NMR (125 MHz, CDCl$_3$)

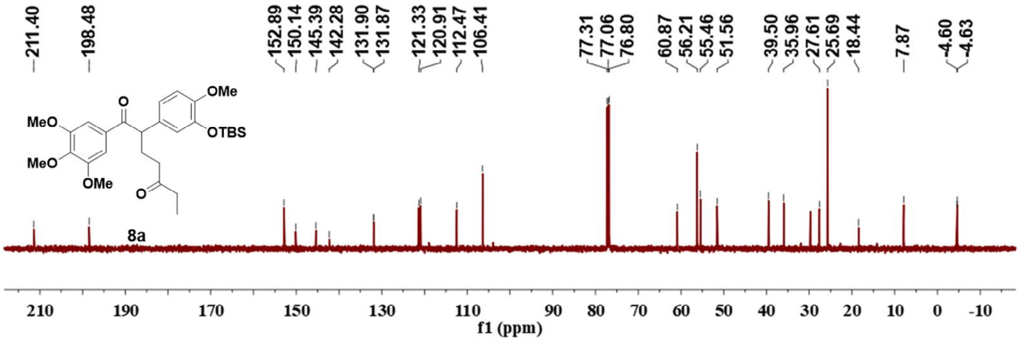

^{13}C DPET135

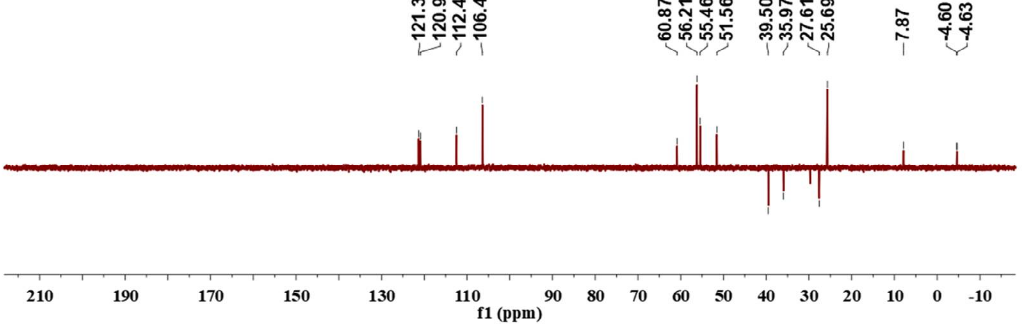

Figure S10. ^{1}H, DEPT135 and ^{13}C NMR ($CDCl_3$) spectra of compound **9**.

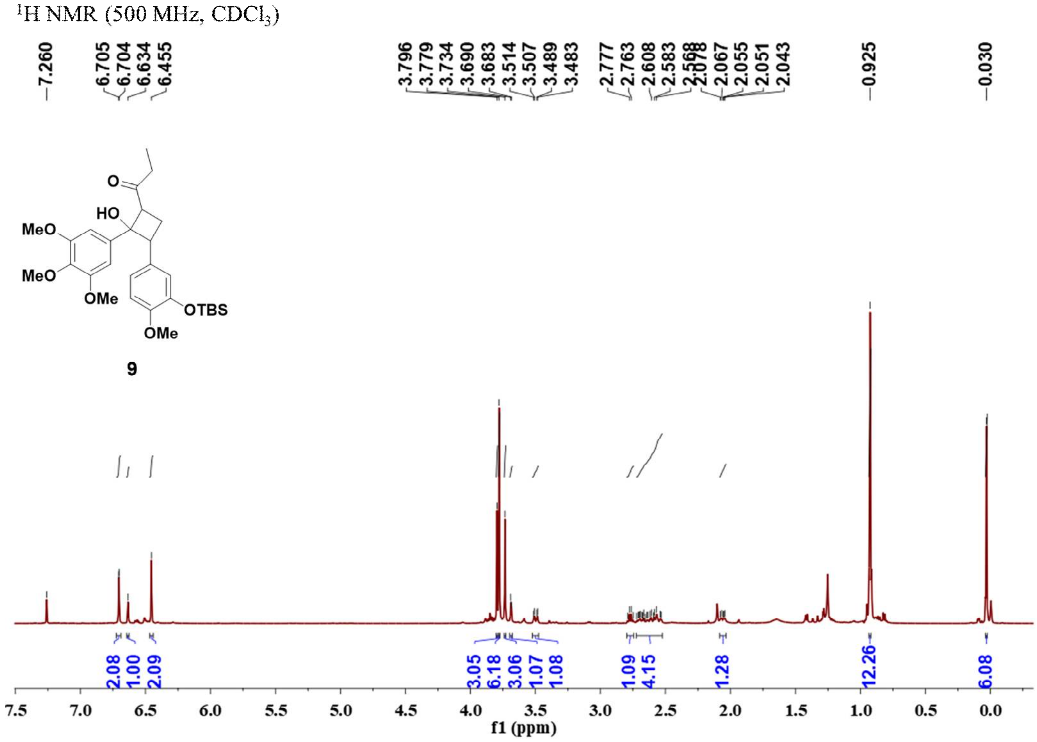

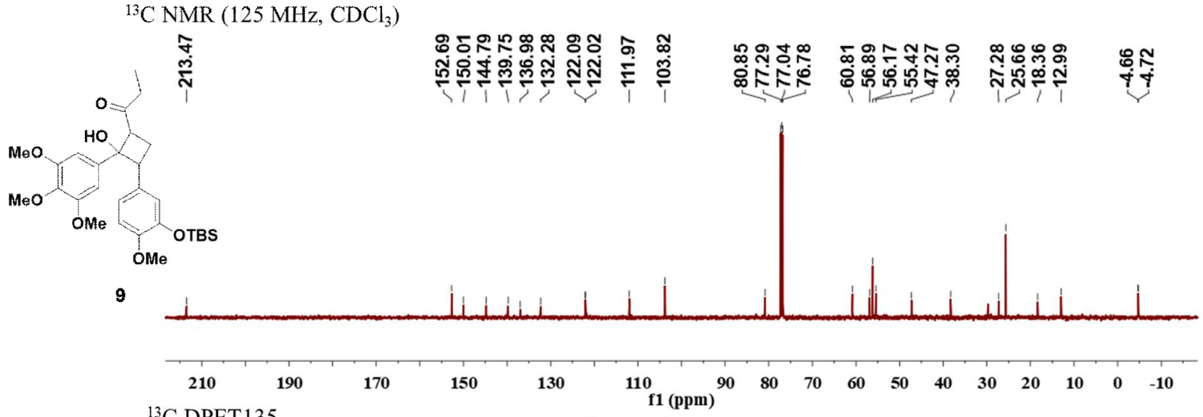

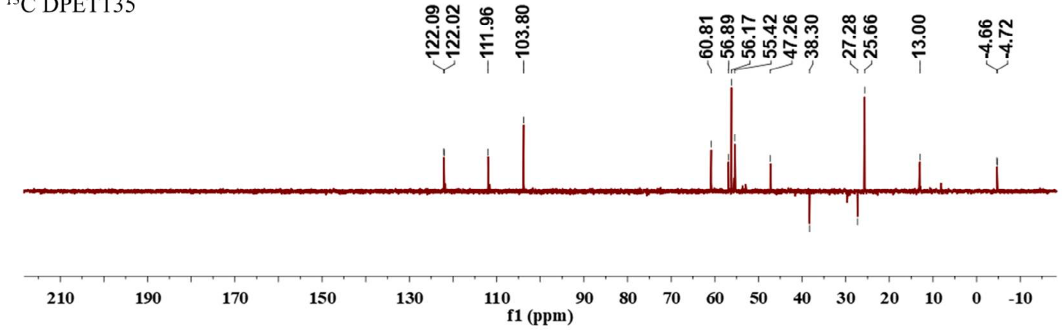

Figure S11. ^{1}H, DEPT135 and ^{13}C NMR ($CDCl_3$) spectra of compound **10a**.

^{1}H NMR (500 MHz, $CDCl_3$)

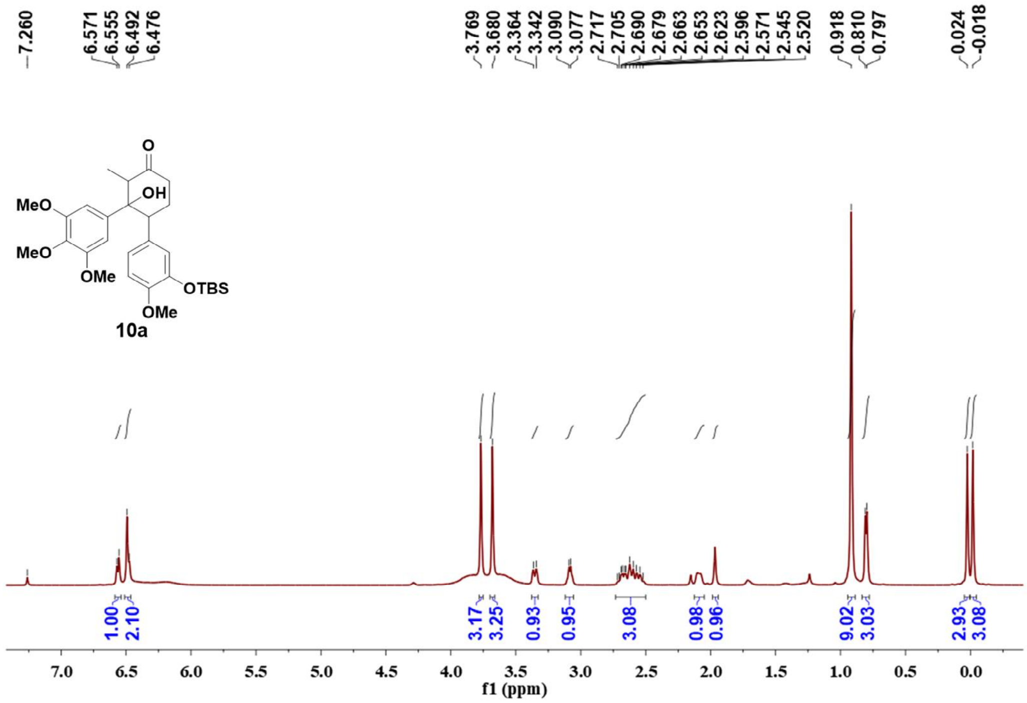

^{13}C NMR (125 MHz, $CDCl_3$)

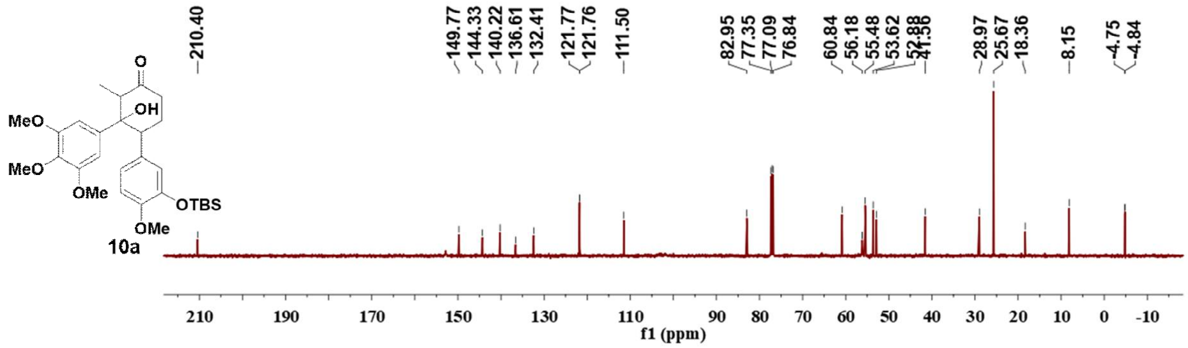

^{13}C DPET135

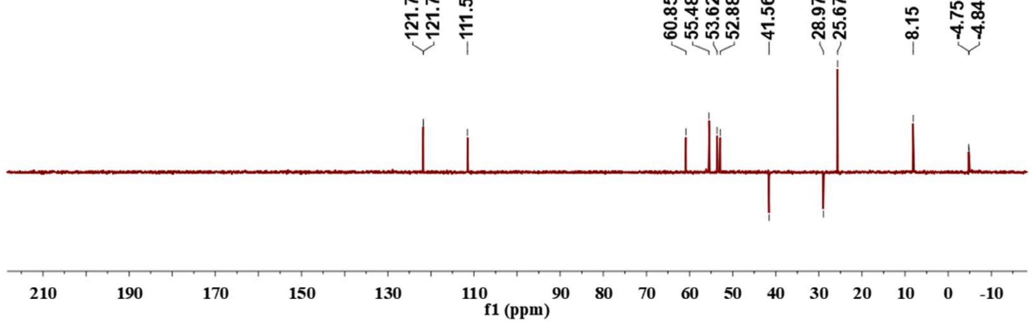

Figure S12. ^{1}H, DEPT135 and ^{13}C NMR (DMSO-d_6) spectra of compound **10b**.

^{1}H NMR (500 MHz, DMSO-d6)

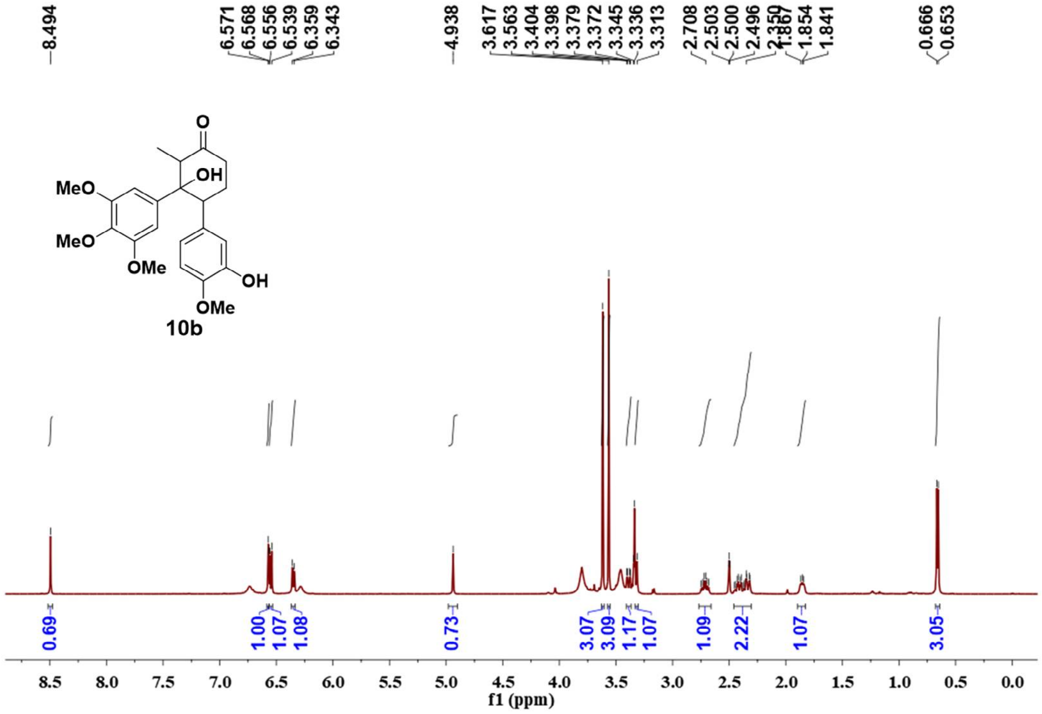

^{13}C NMR (125 MHz, DMSO-d6)

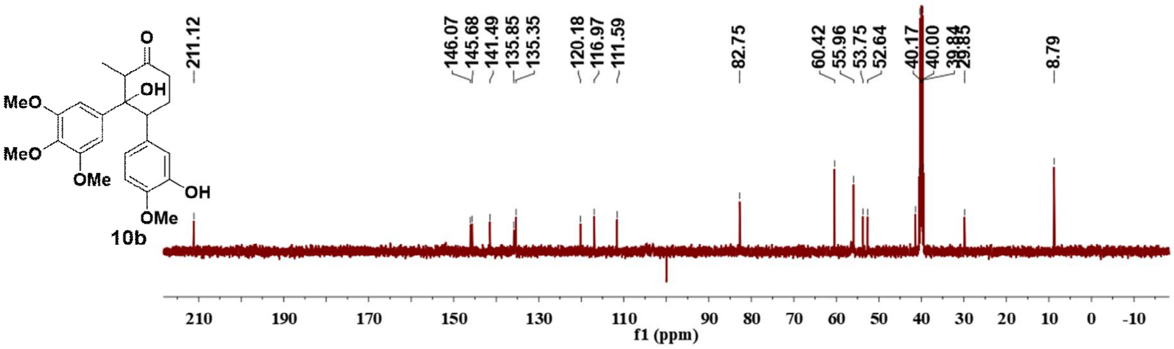

^{13}C DPET135

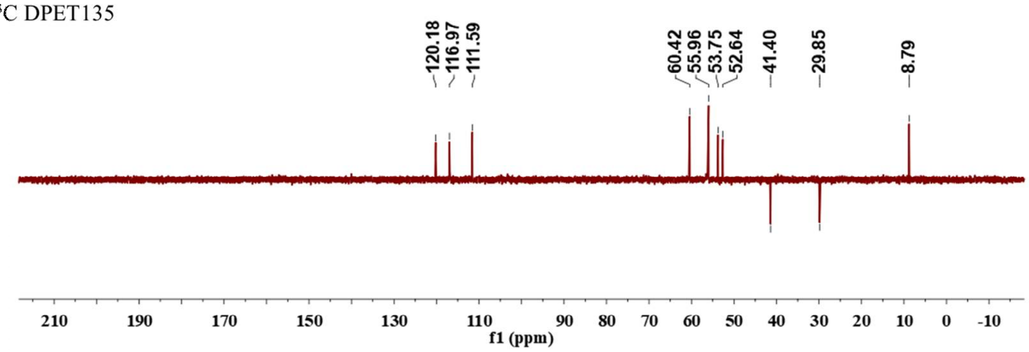

Figure S13. ^{1}H, DEPT135 and ^{13}C NMR ($CDCl_3$) spectra of compound **11**.

^{1}H NMR (500 MHz, $CDCl_3$)

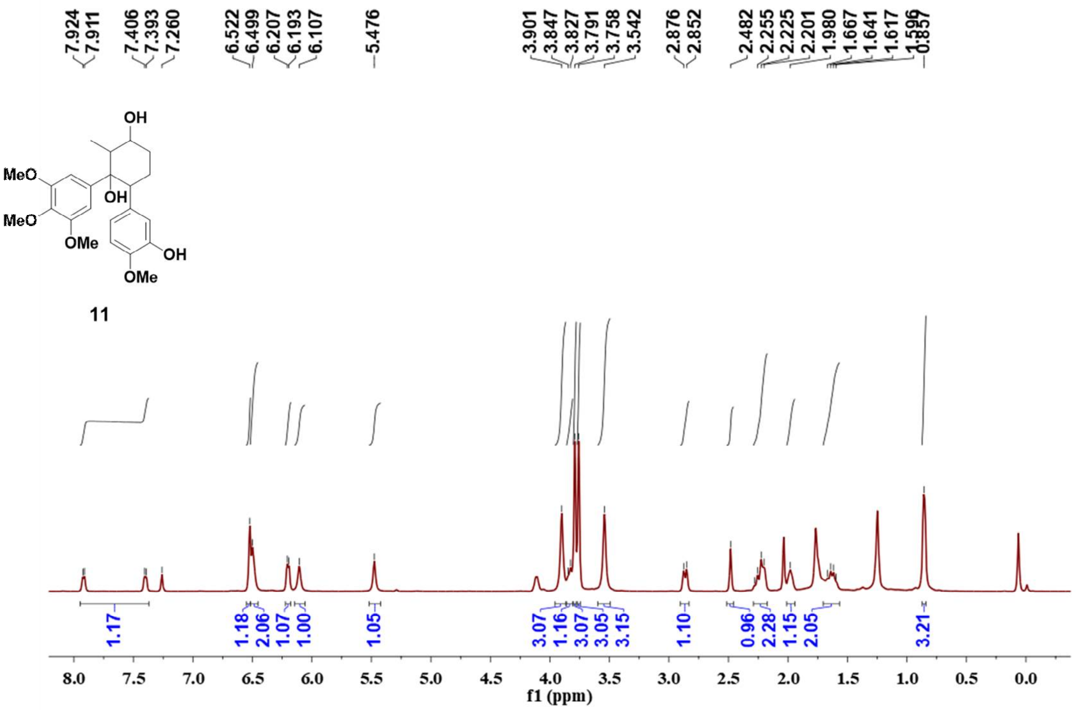

^{13}C NMR (125 MHz, $CDCl_3$)

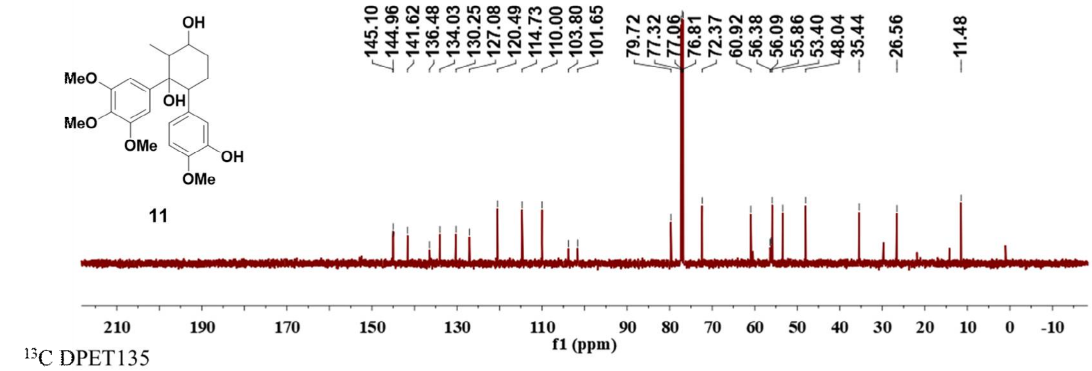

^{13}C DPET135

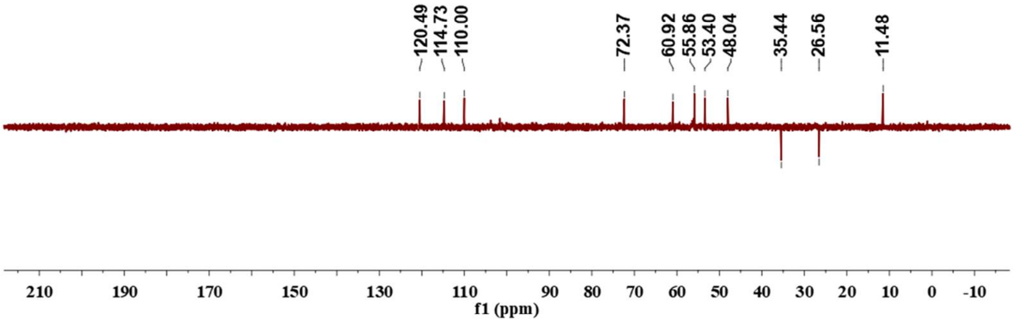

Figure S14. ^{1}H, DEPT135 and ^{13}C NMR ($CDCl_3$) spectra of compound **12a**.

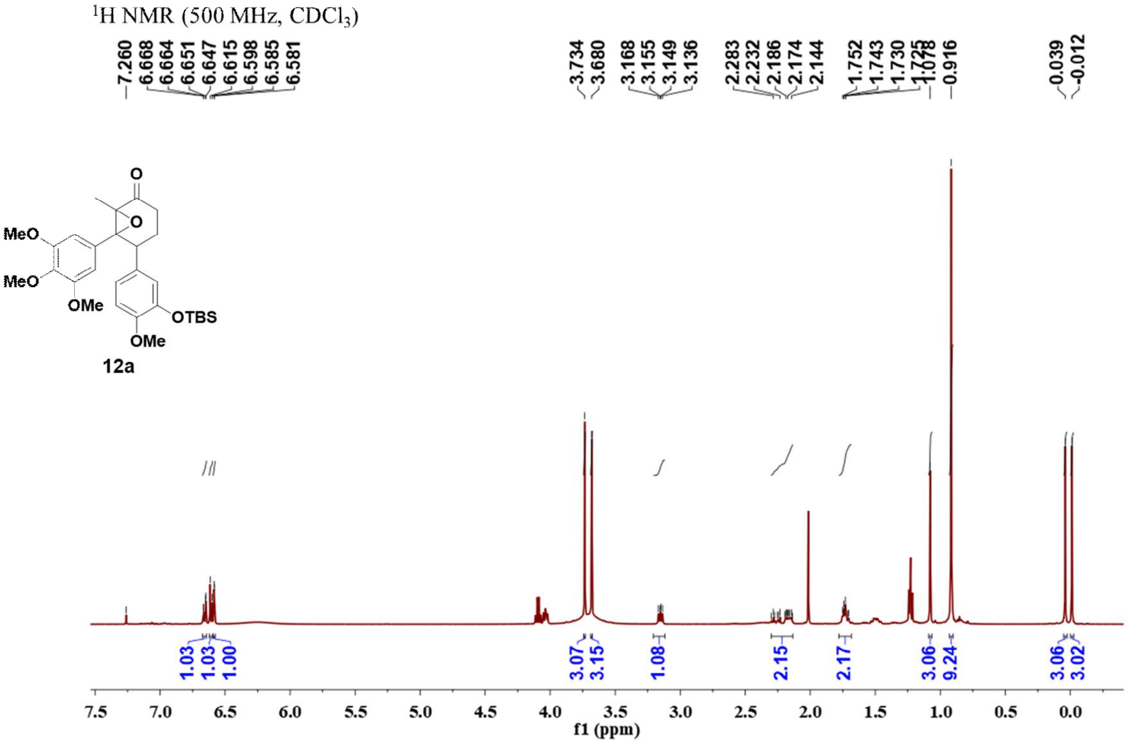

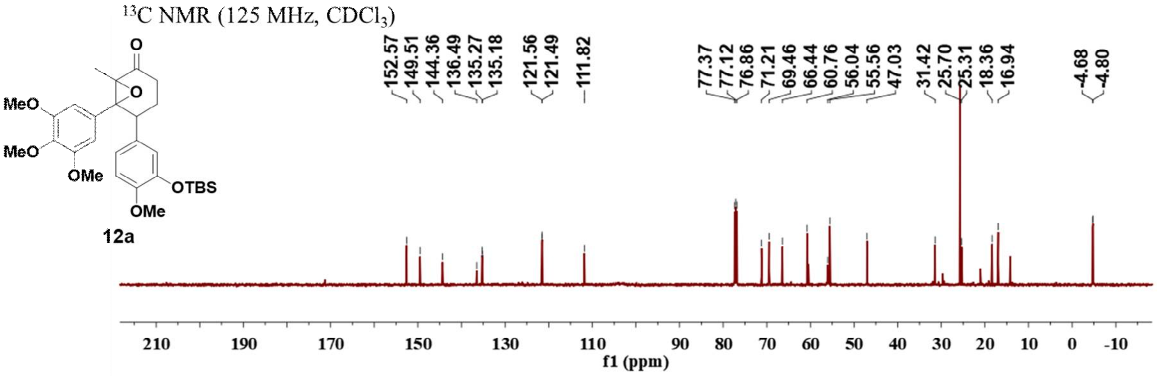

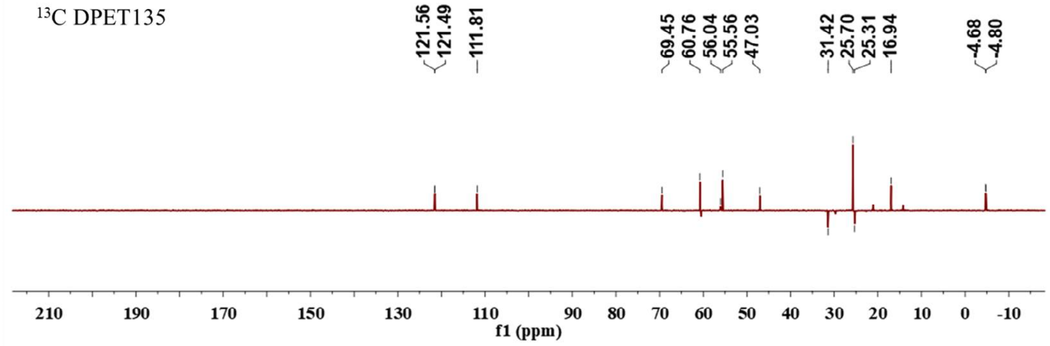

Figure S15. ^{1}H, DEPT135 and ^{13}C NMR ($CDCl_3$) spectra of compound **12**.

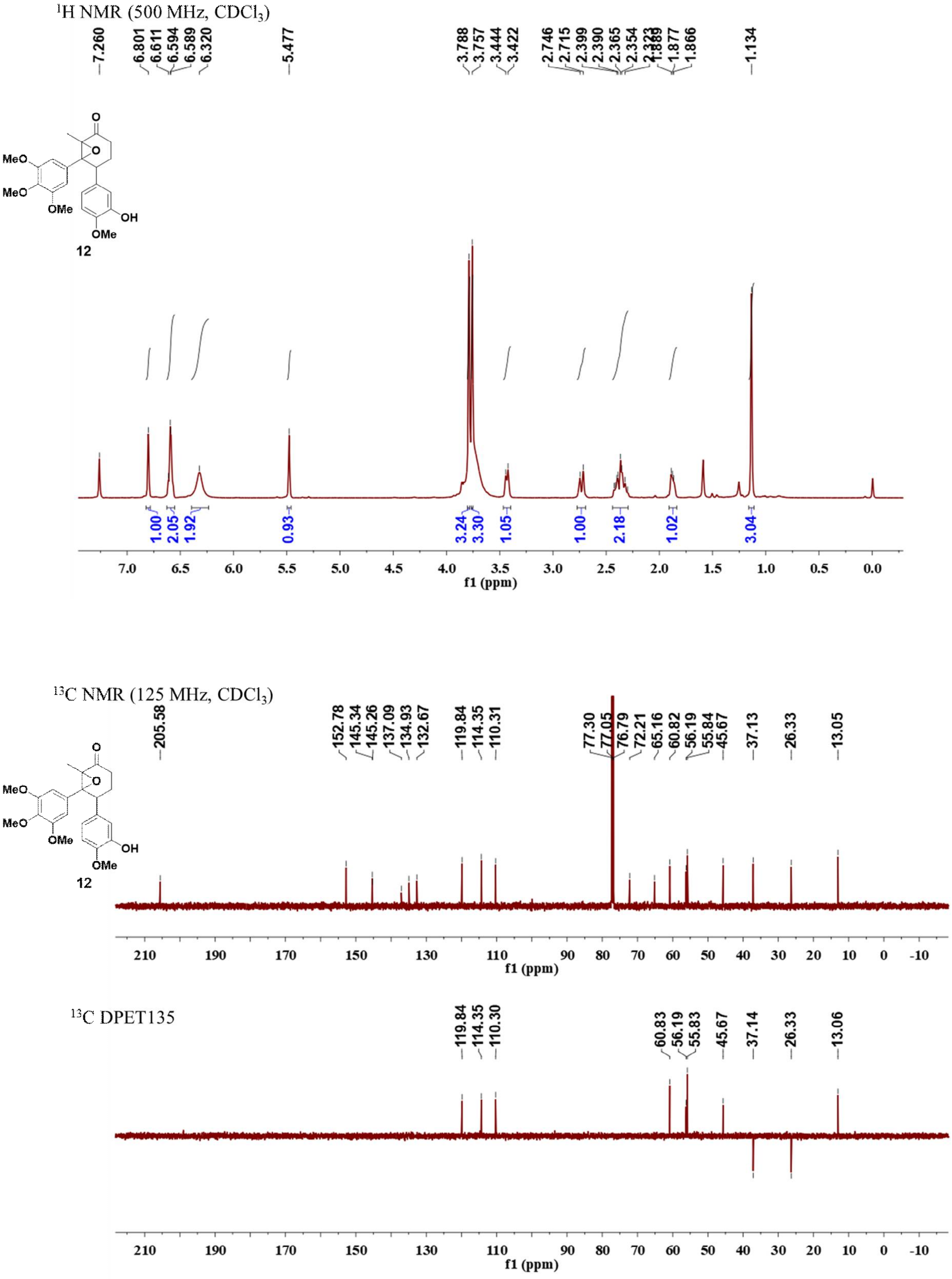

Figure S16. HPLC preparation of **6b-(*Z*)** and **6b-(*E*).** HPLC preparation was performed on Waters 515-2996 with a column of ODS-C18 (2.5 μm, 10 mm× 250 mm).

6b-(*Z*): t_R = 18.639 min, purity = 90.4% at 220 nm, 30 min gradient, 40% MeOH: 60% H_2O to 100% MeOH.

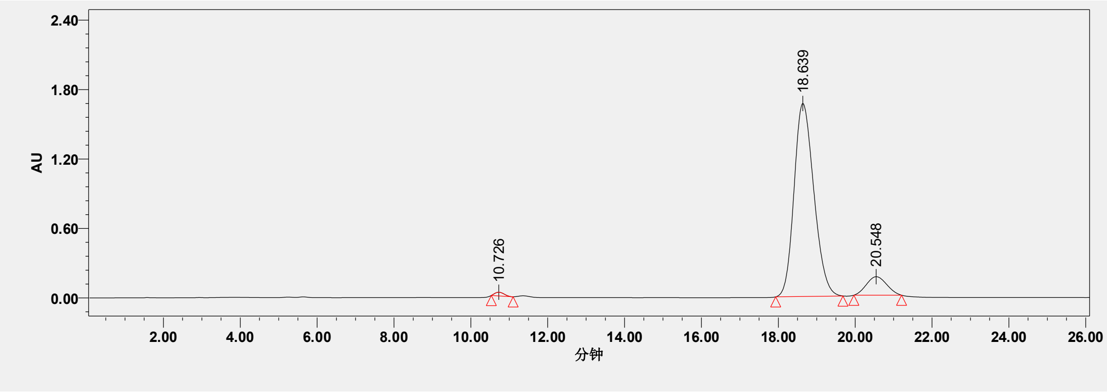

6b-(*E*): t_R = 20.559 min, purity = 91.8% at 220 nm, 30 min gradient, 40% MeOH: 60% H_2O to 100% MeOH.

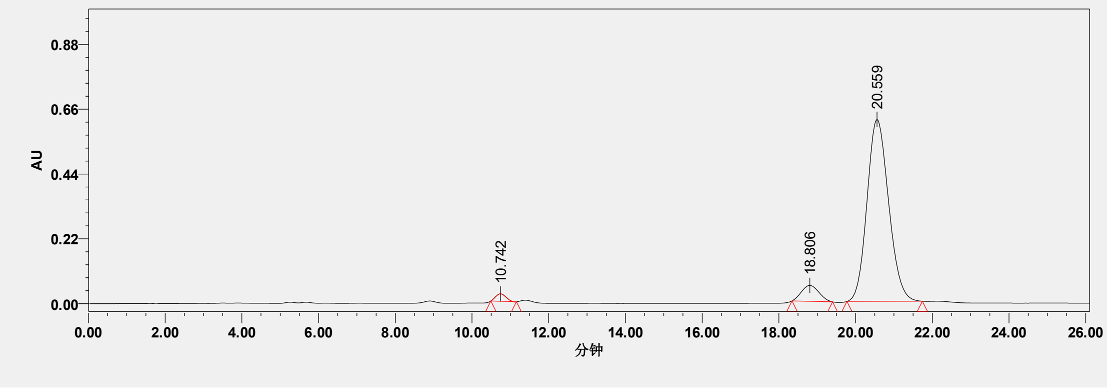

Supplement: Supplementary file 1 [file ijms-21-01817-s001.pdf]
